# Integrated machine learning survival framework develops a prognostic model based on inter-crosstalk definition of mitochondrial function and cell death patterns in a large multicenter cohort for lower-grade glioma

**DOI:** 10.1186/s12967-023-04468-x

**Published:** 2023-09-02

**Authors:** Hu Qin, Aimitaji Abulaiti, Aierpati Maimaiti, Zulihuma Abulaiti, Guofeng Fan, Yirizhati Aili, Wenyu Ji, Zengliang Wang, Yongxin Wang

**Affiliations:** 1 Department of Neurosurgery, Neurosurgery Centre, The First Affiliated Hospital of Xinjiang Medical University, No. 137, South Liyushan Road, Xinshi District, Urumqi City, 830054 Xinjiang China; 2 Department of Obstetrics and Gynecology, The First Affiliated Hospital of Xinjiang Medical University, Urumqi, 830054 Xinjiang China

**Keywords:** Machine learning, Precision oncology, Lower-grade glioma, Programmed cell death, Mitochondrial function

## Abstract

**Background:**

Lower-grade glioma (LGG) is a highly heterogeneous disease that presents challenges in accurately predicting patient prognosis. Mitochondria play a central role in the energy metabolism of eukaryotic cells and can influence cell death mechanisms, which are critical in tumorigenesis and progression. However, the prognostic significance of the interplay between mitochondrial function and cell death in LGG requires further investigation.

**Methods:**

We employed a robust computational framework to investigate the relationship between mitochondrial function and 18 cell death patterns in a cohort of 1467 LGG patients from six multicenter cohorts worldwide. A total of 10 commonly used machine learning algorithms were collected and subsequently combined into 101 unique combinations. Ultimately, we devised the mitochondria-associated programmed cell death index (mtPCDI) using machine learning models that exhibited optimal performance.

**Results:**

The mtPCDI, generated by combining 18 highly influential genes, demonstrated strong predictive performance for prognosis in LGG patients. Biologically, mtPCDI exhibited a significant correlation with immune and metabolic signatures. The high mtPCDI group exhibited enriched metabolic pathways and a heightened immune activity profile. Of particular importance, our mtPCDI maintains its status as the most potent prognostic indicator even following adjustment for potential confounding factors, surpassing established clinical models in predictive strength.

**Conclusion:**

Our utilization of a robust machine learning framework highlights the significant potential of mtPCDI in providing personalized risk assessment and tailored recommendations for metabolic and immunotherapy interventions for individuals diagnosed with LGG. Of particular significance, the signature features highly influential genes that present further prospects for future investigations into the role of PCD within mitochondrial function.

**Supplementary Information:**

The online version contains supplementary material available at 10.1186/s12967-023-04468-x.

## Introduction

Gliomas are the majority prevalent primary tumors that develop of the nervous system in the brain [1]. The classification of gliomas by the WHO is based on histological differences, resulting in four grades, with WHO II and III being classified as lower-grade gliomas (LGG). LGG have a more favorable prognosis compared to glioblastoma (GBM) [2]. While surgery is the recommended treatment for LGG, the invasive nature or close proximity of these tumors to critical tissues can make complete removal challenging. The current standard treatment for LGG involves surgery followed by radiotherapy, with chemotherapy serving as a promising alternative therapy [3]. Unfortunately, the findings suggest that all LGG survivors, regardless of their treatment approach (surgical only management or no treatment), are at risk of experiencing long-term cognitive impairments in various domains [4]. Despite the current standard treatment, which yields a median survival time of 5–10 years for LGG patients [5]. Hence, there is a pressing need to unravel the underlying molecular mechanisms and develop a dependable molecular classification model that can effectively evaluate prognosis and guide personalized treatment strategies for individuals diagnosed with LGG.

Programmed cell death (PCD) is a crucial physiological process that plays a pivotal role in maintaining tissue homeostasis and eliminating damaged or unwanted cells. PCD can occur through various mechanisms, including apoptosis, anoikis, autophagy, alkaliptosis, cuproptosis, entosis, entotic cell death, immunogenic cell death, ferroptosis, lysosome-dependent cell death, methuosis, necroptosis, netoticcelldeath, NETosis, oxeiptosis, pyroptosis, parthanatos, and paraptosis [6]. Imagine PCD as a housekeeping system within the body. Just like we clean our houses to maintain cleanliness and order, PCD acts as an internal cleaning mechanism that removes damaged or unnecessary cells to keep the tissues healthy. Among these PCD mechanisms, mitochondrial dysfunction has been implicated in several of them [7]. Mitochondria play a pivotal role in supplying energy for cellular functions, regulating cellular signaling pathways, and governing PCD. Studies have shown that mitochondrial dysfunction, characterized by changes in mitochondrial structure, function, and dynamics, is associated with decreased mitochondrial respiration, altered mitochondrial morphology, and impaired mitochondrial quality control in LGG [8–10]. Apoptosis is a widely recognized mechanism of PCD, which serves an essential function in preserving tissue homeostasis and eliminating damaged or unnecessary cells. Apoptosis is typified by a sequence of biochemical and morphological alterations [11, 12]. Pyroptosis is a form of PCD that occurs following inflammasome activation and caspase-1 cleavage. Cellular enlargement, membrane rupture, and the production of pro-inflammatory cytokines are its defining features [13]. Ferroptosis is a recently identified type of PCD defined by iron-dependent cellular demise and lipid peroxidation [14]. Autophagy is a cellular mechanism that is essential for preserving cellular equilibrium by breaking down impaired proteins and organelles. Autophagy can serve as a mechanism for either promoting cell survival or inducing cell death, depending on the specific context in which it occurs [15]. Necroptosis is a form of PCD that is characterized by necrosis-like cell death and is triggered by the activation of RIPK1 and RIPK3 [6]. Cuproptosis is a form of PCD that is triggered by copper overload and is characterized by lipid peroxidation and mitochondrial dysfunction. Entotic cell death occurs exclusively in viable cells and their adjacent regions. Unlike the traditional apoptotic pathway, entotic cell death does not require the activation of apoptotic executioner pathways [16]. Netotic cell death is an additional type of PCD that occurs due to the discharge of neutrophil extracellular traps (NETs), commonly observed in response to infections or injuries [17]. Parthanatos is a tightly controlled type of cell death triggered by excessive activation of the nuclease PARP-1 [18]. The process of lysosome-mediated cell death involves the action of hydrolases that enter the cytosol through membrane permeabilization [19]. Additionally, alkaliptosis, an emerging type of programmed cell death, is controlled by the process of intracellular alkalinization [20]. Oxeiptosis, which utilizes the reactive oxygen sensing capabilities of KEAP1, is a recently discovered cellular pathway that is likely to operate in conjunction with other cell death pathways [21].

In the context of LGG, tumor cells can evade PCD mechanisms through various strategies, similar to concealing garbage in hidden corners of a room. They may change their shape or activate specific signaling pathways to escape elimination. The increased understanding of PCD mechanisms has led to the development of numerous drugs that target these pathways. For instance, the FDA approved a BCL-2 inhibitor that regulates cell apoptosis, which is effective in treating lymphoma [22]. GSDME-induced pyroptosis, a distinct form of programmed cell death, has shown potential as an anti-tumor immunotherapy [23]. Furthermore, research has demonstrated that obstructing ferroptosis can trigger cellular resistance to anti-PD-1/PD-L1 treatment [24]. These findings demonstrate the importance of PCD research in advancing our understanding of LGG and developing new therapies to combat them.

Disrupted mitochondrial morphology, such as changes in shape, size, or cristae organization, can disrupt normal mitochondrial function and trigger PCD [25–27]. Structural abnormalities may affect the release of pro-apoptotic factors from the mitochondria, leading to caspase activation and subsequent apoptosis [27]. Mitochondrial function is also closely linked to PCD mechanisms. Dysfunctional mitochondria with impaired oxidative phosphorylation and ATP production can induce cellular stress and initiate PCD pathways [25]. To better link mitochondrial function to PCD as described above, a series of related biomarkers were screened in this study. Nowadays, several molecular markers have been identified as clinically significant in both the diagnosis and prognosis of LGG [28–31]. Markers such as IDH1 mutation and MGMT promoter methylation play a critical role in determining the optimal postsurgical treatment, including adjuvant chemotherapy and radiotherapy, and are strongly associated with the prognosis of LGG [32]. In addition to genetic factors, various environmental and lifestyle factors can contribute to the development of LGG [33]. The carcinogens present in tobacco smoke have the potential to inflict DNA damage on brain cells, thus facilitating tumor formation [34]. There have been studies examining the potential correlation between exposure to electromagnetic fields (EMF), such as those emitted by power lines or electronic devices, and the risk of brain tumors, including LGG [35].

In the process of screening and proving numerous biomarkers, sources of bias such as sample selection bias, tumor heterogeneity, analytical bias, and publication bias can impact the accuracy and generalizability of the results [36]. Hence, the identification of survival-associated genes through transcriptome-based databases is necessary for prognostic prediction and targeted treatment selection. For decades, researchers have demonstrated that mitochondrial dysfunction and PCD mechanisms are essential for the development and spread of malignant neoplasms. In order for malignant cells to progress, they must overcome various forms of cell death and mitochondrial dysfunction. Nevertheless, there is still a lack of comprehensive understanding regarding the interplay between mitochondrial dysfunction and PCD in LGG, and there are limited detailed functional studies of these processes in LGG. To fill these knowledge gaps, we introduced a novel metric called the mitochondrial programmed cell death index (mtPCDI) to forecast the efficacy and prognosis of therapeutic interventions in LGG. Through our investigation, we discovered the heterogeneity among LGG patients and evaluated their clinical outlook. While our findings rely on the hypothesis of an interaction between mitochondrial dysfunction and PCD, this provides valuable guidance for selecting the best treatment options.

## Materials and methods

### Data collection

We obtained clinical details and transcriptome data of individuals suffering from LGG from four databases: the Cancer Genome Atlas (TCGA, https://portal.gdc.cancer.gov), Chinese Glioma Genome Atlas (CGGA, http://www.cgga.org.cn/), Gene Expression Omnibus (GEO, http://www.ncbi.nlm.nih.gov/geo), and ArrayExpress (https://www.ebi.ac.uk/biostudies/arrayexpress). The total analysis included 1467 samples, with 506 samples from TCGA-LGG, 172 samples from CGGA-325, 420 samples from CGGA-693, 121 samples from Rembrandt, 121 samples from GSE16011, and 142 samples from E-MTAB-3892. To improve comparability across datasets, all RNA-seq data were converted to transcripts per million (TPM) format and corrected for batch effects using the “combat” function of the “sva” package. Prior to analysis, all data were log-transformed.

In data collection for differential expression analysis, we acquired RNAseq data in TPM (Transcripts Per Million) format from two distinct sources: the TCGA and the Genotype-Tissue Expression (GTEx) project. Specifically, we retrieved 506 LGG samples (WHO grade II and III) from TCGA and 105 normal cerebral cortex samples from GTEx. For consistency and standardized processing, all the data underwent uniform processing using the Toil process [37] from UCSC XENA (https://xenabrowser.net/datapages/). Harmonized data processing ensures that the datasets are compatible and reduces any potential bias arising from variations in data preprocessing methods.

### Identification of prognostic mitochondria-related genes and PCD-related genes

We conducted a literature search [38] and gathered 18 patterns of PCD and key regulatory genes, which included 580 genes related to apoptosis, 367 genes related to autophagy, 7 genes related to alkaliptosis, 338 genes related to anoikis, 19 genes related to cuproptosis, 15 genes related to enteric cell death, 87 genes related to ferroptosis, 34 genes related to immunogenic cell death, 220 genes related to lysosome-dependent cell death, 101 genes related to necroptosis, 8 genes related to netotic cell death, 24 genes related to NETosis, 5 genes related to oxeiptosis, 52 genes related to pyroptosis, 9 genes related to parthanatos, and 66 genes related to paraptosis. Additionally, there were 8 Methuosis genes and 23 Entosis genes, resulting in a total of 1964 PCD-related genes. We removed 416 duplicates, resulting in 1548 PCD-related cluster genes for our analysis (Additional file 9: Table S1). From MitoCarta 3.0 [39], we extracted 1136 mitochondria-related genes (Additional file 10: Table S2).

We employed the “limma” package to identify genes with differential expression in LGG and their associated normal tissues. To determine differential expression, we set thresholds of log2 fold change (log2FC) greater than 2 and a false discovery rate (FDR) less than 0.05. Subsequently, we utilized the “VennDiagram” package to show visual representations in differentially expressed genes (DEGs) related to mitochondrial function and programmed cell death. Additionally, we conducted Pearson correlation analysis on the RNA-seq data of TCGA-LGG samples to identify mtPCD (mitochondrial programmed cell death) co-expressed genes that exhibit a correlation coefficient (R) greater than 0.6 and a p-value (P) less than 0.001.

### Development of prognostic model

We integrated ten diverse machine learning algorithms and evaluating 101 algorithmic combinations [40, 41]. These machine learning algorithms included Support Vector Machine (SVM), Least Absolute Shrinkage and Selection Operator (Lasso), Gradient Boosting Machine (GBM), Random Forest, Elastic Net, Stepwise Cox, Ridge, CoxBoost, Super Partial Correlation (SuperPC), and Partial Least Squares with Cox regression (plsRcox). We followed a sequential approach [40] that involved identifying prognostic variables using univariate Cox regression modeling, developing prediction models on the TCGA-LGG cohort, validating these models on five external and independent datasets (CGGA-325, CGGA-693, Rembrandt, GSE16011, and E-MTAB-3892), and calculating the Harrell Consistency Index (C-index) for model selection. We defined the model with the highest average C-index in all cohorts as the optimal model. Based on previous descriptions in the references [40, 42], we categorized LGG patients into high- and low-mtPCDI cohorts using the median score of the respective cohort. Subsequently, we assessed the prognostic significance using Kaplan–Meier curves. Additionally, Calibration curves and Receiver Operating Characteristic (ROC) curves were generated to evaluate the mtPCDI's prognostic efficacy.

### Biological function and pathway enrichment analysis

To identify genes that exhibited significant differential expression between the high and low mtPCDI categories, we applied selection criteria of FDR < 0.05 and log2 fold change (FC) > 1. In order to explore the biological functions and pathway processes associated with mtPCDI, we conducted gene ontology (GO) and Kyoto Encyclopedia of Genes and Genomes (KEGG) analysis using “clusterProfiler” package. Briefly, we used the above differential genes as inputs, converted them to Entrez id before GO and KEGG enrichment analysis, and used a adj. p-value < 0.05 as a criterion. Gene set enrichment analyses (GSEA) enrichment were conducted for differential genes between different mtPCDI groups. In addition, Gene Set Variation Analysis (GSVA) was performed to investigate the heterogeneity of various biological processes, using the “GSVA” package [43].

### Assessment of the immune microenvironment

To assess the degree of immunological infiltrate comprehensively, we utilized various bioinformatic algorithms, including single-sample Gene Set Enrichment Analysis (ssGSEA) [44], Tumor Immune Estimation Resource (TIMER) [45], Cell-type Identification by Estimating Relative Subpopulations of RNA Transcripts (CIBERSORT) [46], CIBERSORT-ABS [47], QUANTISEQ [48], Microenvironment Cell Populations-counter (MCP-counter) [49], Xcell [50], and Estimation of Proportion of Immune and Cancer cells (EPIC) [51]. Each algorithm employed unique strategies and gene expression signatures to estimate the abundance of different immune cell subpopulations. By computing the enrichment or relative abundance of marker genes, we accurately estimated the proportions of invading immune cell types in the LGG samples. Furthermore, we employed the “Estimation of Stromal and Immune cells in Malignant Tumor tissues using Expression data” (ESTIMATE) algorithm to generate overall immunological scores. Finally, we conducted an analysis of the expression patterns of 60 immunomodulatory genes [52], including those involved in antigen presentation, cell adhesion, co-inhibitors, co-stimulators, ligands, and receptors. Finally, we utilized the Wilcoxon signed rank summation test. This statistical test allowed us to determine the significance of differences in immune infiltration. Additionally, we generated heat maps showing the abundance of immune infiltration for each LGG sample under the distinct algorithms used, providing a visual representation of these differences.

### Development of nomogram scoring system

We employed the ‘rms’ package, a widely used tool in statistical analysis and modeling, to develop a nomogram. This nomogram aimed to integrate clinical and pathological features with mtPCDI to predict patient survival. Nomograms are graphical predictive models that provide a personalized probability of an outcome based on individual characteristics. To validate the precision of the projected survival rates at 1-, 3-, and 5-year intervals, we generated calibration plots.

### The mtPCDI signature’s function in forecasting drug susceptibility

We utilized the ‘pRRophetic’ and ‘oncoPredict’ packages to determine the half-maximal inhibitory concentration (IC50) of chemotherapy drugs commonly administered for LGG treatment in both the high- and low-mtPCDI groups.

### Cluster analysis of mtPCDI signature genes

We performed a consensus cluster analysis on tumor samples using the expression levels of 18 mtPCDI marker genes, employing the “ConsensusClusterPlus” package. The objective of this analysis was to classify the samples into distinct subgroups. In addition, we employed t-SNE analysis and principal component analysis (PCA) to visually evaluate the clustering patterns of the samples.

### Pan-cancer analysis of mtPCDI signature genes

For each type of cancer, our analysis compared tumor expression with normal tissue surrounding the tumor. The gene expression patterns of signature genes (log2FC > 1.5, FDR < 0.05) were examined specifically. We utilized clinical data from 33 different cancer tumor samples obtained from TCGA to investigate the relationship between gene expression and survival. Moreover, to assess the impact of gene expression on patient survival, we constructed a survival landscape for the signature-derived genes. We categorized the tumor specimens into low- and high-expression groups based on mRNA values and utilized the “survival” package to analyze the survival times and status within these groups. Furthermore, we examined copy number variation (CNV) data from 11,495 samples within the TCGA database. Our objective was to identify instances of significant CNV amplifications or deletions within the cohort. We considered both amplifications and deletions to enhance the detection of alterations in each gene. We defined high-frequency CNVs as those with a frequency exceeding 5%. Additionally, we collected single nucleotide variant (SNV) data from a total of 10,234 samples across 33 different types of cancers. Our aim was to understand the overall mutation frequencies across pan-cancer. To summarize the SNV data, we generated percentage heat maps. Finally, we compared the methylation patterns of each gene between tumor and normal samples using the Wilcoxon signed rank test. We identified significantly hypo- or hypermethylated genes using a probability value threshold of 0.05.

### Statistical analyses

Statistical analyses were performed to assess the significance of observed differences and correlations in the study. All data were expressed as mean ± SD (standard deviation). To evaluate the impact of risk factors on survival outcomes, Cox regression models and Kaplan–Meier (K-M) survival analysis were utilized. Pearson correlation analysis was conducted to explore the relationships between variables. Statistical analysis and scientific graphing were performed using R Studio (version 4.2.3). A significance level of p < 0.05 was considered statistically significant.

## Results

### Preliminary screening of mtPCDI regulators

**Fig. 1:**
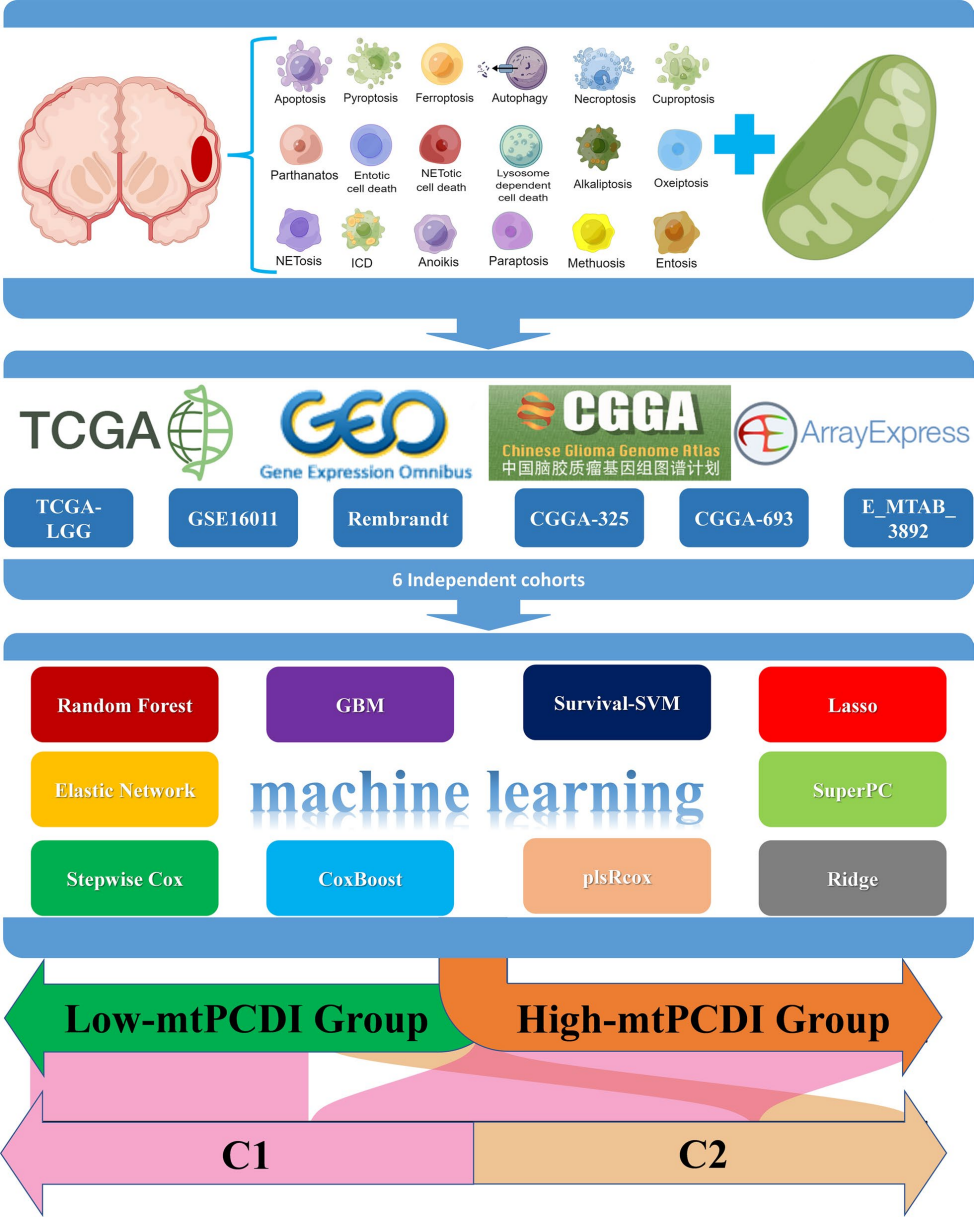
The graphic depicts the study's process

**Fig. 2:**
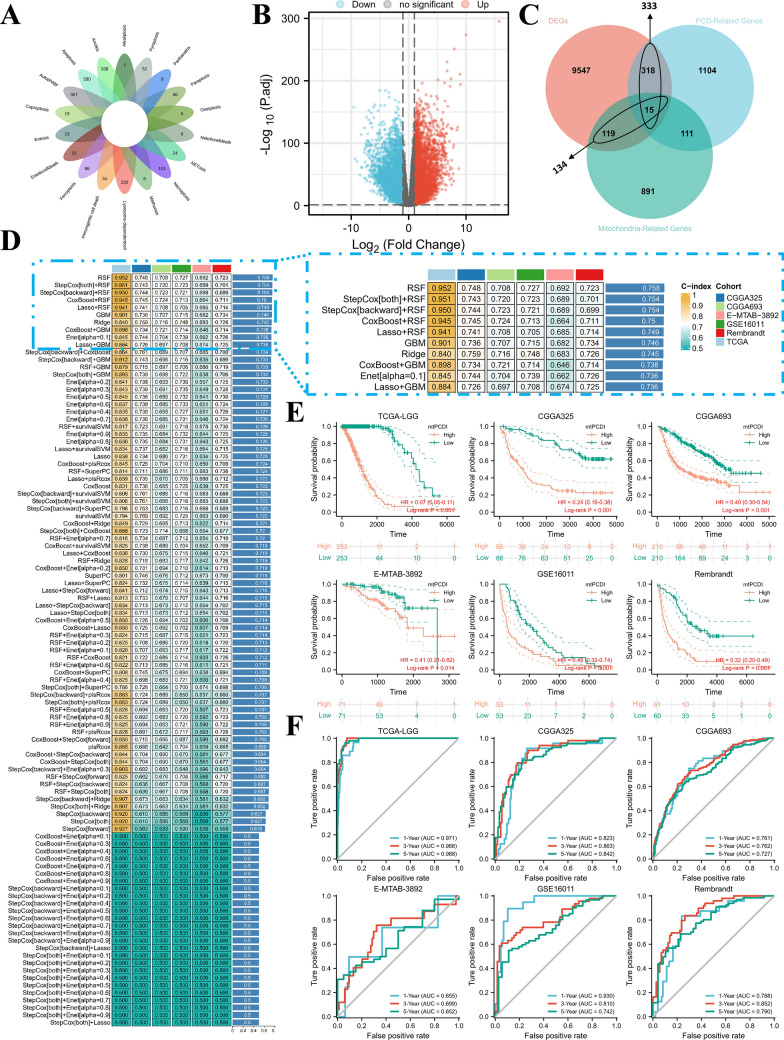
The Prognostic Significance of the mtPCDI. **A** Collection of key regulatory genes containing 18 PCD patterns. **B** Identification of differentially expressed gene volcanoes between cerebral cortex and LGG tissues. **C** 134 mitochondria-associated genes and 333 programmed cell death-associated genes exhibited differential expression in LGG tumour tissues compared to normal tissues. **D** The machine learning algorithms used by mtPCDI were 101 different combinations. Subsequently, each model’s c-index was calculated for each dataset (**E**) TCGA-LGG, CGGA-325, CGGA-693, Rembrandt, GSE16011, and E-MTAB-3892 survival curves by mtPCDI. **F** ROC curves of 1-year, 3-year, and 5-year OS in the each datasets

Our study workflow is depicted in Fig. 1. Initially, we collected essential regulatory genes that encompassed 18 PCD patterns from literature sources [38]. Subsequently, we included 1548 PCD-associated crosstalk genes in our analysis, as illustrated in Fig. 2A. We then performed differential analysis to identify 11,581 genes that exhibited differential expression between normal and tumour tissues, as depicted in Fig. 2B. As demonstrated in Fig. 2C, 134 mitochondria-associated genes and 333 programmed cell death-associated genes exhibited differential expression between different samples. To identify genes involved in mitochondrial and programmed cell death co-crosstalk, applying pearson co-expression (r > 0.6, p < 0.001), a comparison was made between the 333 gene expression patterns associated with programmed cell death and the 134 markers related to mitochondrial function, resulting in 215 mitochondrial function and cell death co-expressed genes (Additional file 11: Table S3). We then applied Cox regression analysis to evaluate whether these 215 co-expressed genes influenced patient prognosis. 146 mtPCD-related genes were revealed in the TCGA-LGG cohort. And exhibited significant associations with overall survival (OS) among patients diagnosed with LGG, as outlined in Additional file 12: Table S4.

### Integrative construction of a consensus signature

We employed ten machine learning algorithms to construct mtPCDI. These algorithms were applied to the TCGA-LGG cohort and five external validation datasets (CGGA-325 and CGGA-693, Rembrandt, GSE16011, and E-MTAB-3892) to determine the optimal model (the largest average C-index in the six cohorts). The final RSF algorithm identified the 18 most valuable mtPCDI signature genes, including ACAA2 (acetyl-CoA acyltransferase 2), ACACB (acetyl-CoA carboxylase beta), ANGPTL2 (angiopoietin-like 2), ANXA5 (annexin A5), BRCA1 (breast cancer type 1 susceptibility protein), BRCA2 (breast cancer type 2 susceptibility protein), CTSL (cathepsin L), ECHDC2 (enoyl-CoA hydratase domain-containing 2), ERCC4 (ERCC excision repair 4), GNS (N-acetylglucosamine-6-sulfatase), IFI16 (interferon gamma-inducible protein 16), MCUB (mitochondrial calcium uniporter), MRPS16 (mitochondrial ribosomal protein S16), MSH6 (MutS Homolog 6), MTPAP (mitochondrial poly(A) polymerase), NCOA4 (nuclear receptor coactivator 4), PABPC5 (poly(A) binding protein cytoplasmic 5), and PDE2A (phosphodiesterase 2A). The best-performing model was constructed (Fig. 2D). Remarkably, in the TCGA-LGG, LGG patients with elevated expression of six signature genes demonstrated an extended survival period, whereas those with increased expression of 12 signature genes had a shortened survival duration (Additional file 1: Figure S1).

Our analysis of patients with LGG in the TCGA-LGG, CGGA-325, CGGA-693, Rembrandt, GSE16011, and E-MTAB-3892 datasets showed that high mtPCDI expression was associated with reduced survival times, as illustrated in Fig. 2E. The TCGA-LGG dataset showed AUC values of 0.971, 0.988, and 0.988 for the three respective years. The CGGA-325 dataset exhibited AUC values of 0.823, 0.863, and 0.842, while the CGGA-693 dataset presented AUC values of 0.761, 0.762, and 0.727. Further, the E-MTAB-3892 dataset displayed AUC values of 0.655, 0.699, and 0.652, and the GSE16011 dataset depicted AUC values of 0.930, 0.810, and 0.742. Lastly, the Rembrandt dataset demonstrated AUC values of 0.788, 0.852, and 0.790 for the same duration. These results underscore the prognostic significance of mtPCDI, as illustrated in Fig. 2F.

### Characterisation of clinical variables

**Fig. 3:**
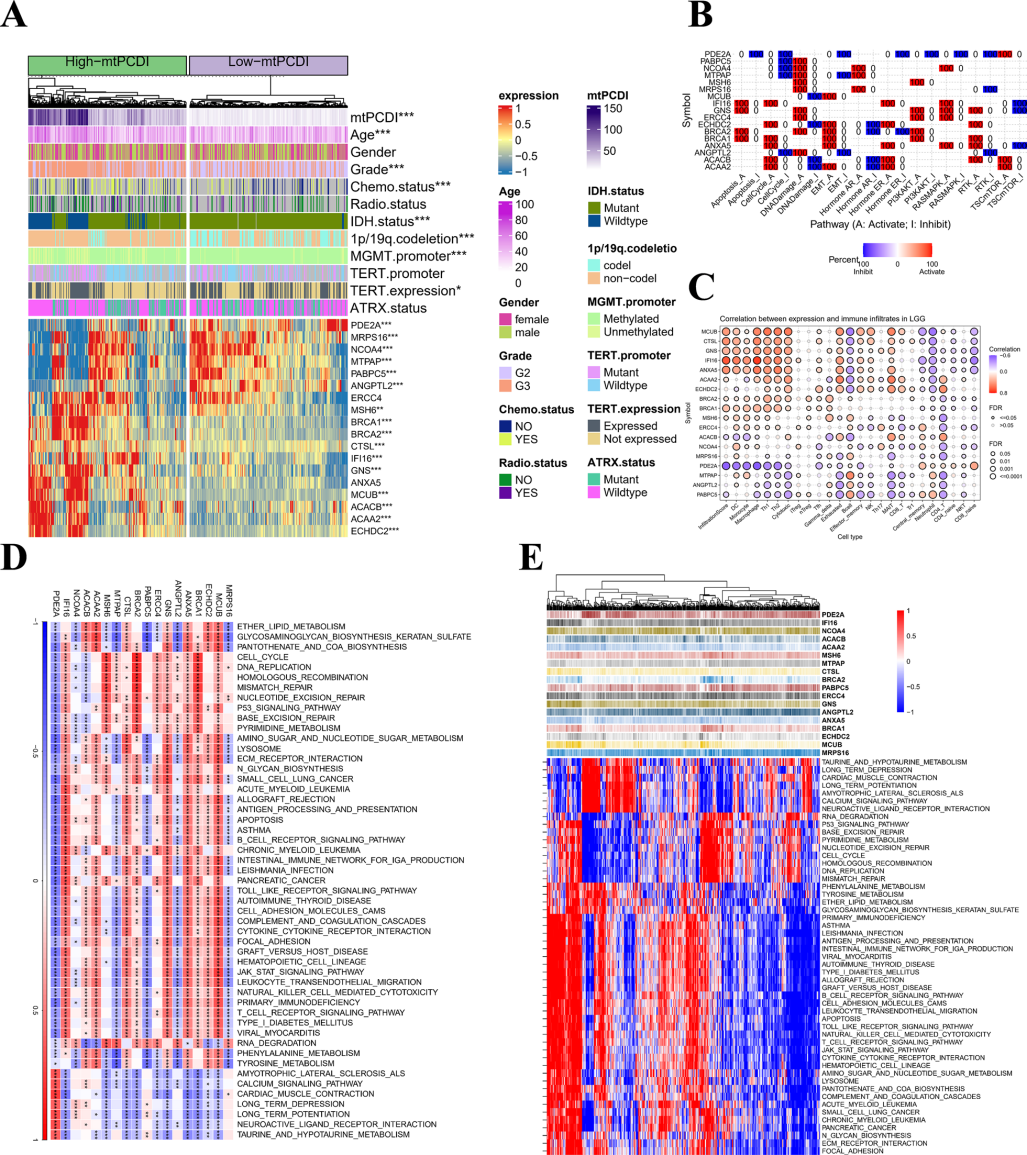
Annotation of characteristics for the mtPCDI signature genes. **A** Heatmap is presented to display the relationship of mtPCDI groups and clinical features, as well as the expression of the 18 most significant genes in patients with LGG. **B** Heatmap displays the proportion of cancers in LGG in which a gene exhibits a significant impact (with FDR ≤ 0.05) on the pathway, with each cell representing the corresponding percentage value. **C** Bubble plot that presents the summary of the correlation between18 mtPCDI signature genes expression and 24 immune cell types infiltrates in LGG. **D** Heat map showing the gene-pathways involved in 18 mtPCDI signature genes. **E** Heatmap displaying the enrichment scores of important pathways. *p < 0.05; **p < 0.01; ***p < 0.001; ns, no statistical significance

To evaluate the predictive performance of our prognostic model for OS in patients with LGG, we categorized LGG patients based on several number of diagnostic features. These include age, gender, tumor level, response to radiation and chemotherapy, IDH1 mutation status, MGMT promoter methylation position, 1p/19q teamed up-deletion situation, TERT expression and TERT mutant status, as well as ATRX mutation status, as described in Additional file 14: Table S6. We compared the mtPCDI between different subgroups of clinical pathological variables and observed significant differences in age stratification, tumor grade, chemotherapy status, 1p/19q co-deletion, IDH1 mutation status and MGMT inducer methylation position (p < 0.05) among different subgroups, as illustrated in Additional file 2: Figure S2. Moreover, Kaplan–Meier analysis demonstrated that the OS frequency was greater in the lower mtPCDI cohort contrasted to the higher mtPCDI category, as illustrated in Additional file 3: Figure S3. The heatmap of the mtPCDI prognostic model and clinical pathological variables is depicted in Fig. 3A.

### Annotation of characteristics for the mtPCDI signature genes

We conducted a comprehensive analysis of the somatic mutation frequency of the 18 mtPCDI signature genes, revealing that their mutation frequency was remarkably low in TCGA-LGG samples. Of the signature genes, ACACB and BRCA2 exhibited the highest mutation frequency at a mere 2% (Additional file 4: Figure S4A). To obtain more detailed information, we looked at where the 18 mtPCDI signature genes—which are found on chromosomes—had CNV changes (Additional file 4: Figure S4B), and found their ubiquity upon further analysis of CNV frequency (Additional file 4: Figure S4C). We found that there was a substantia variation in the expression of 16 signature genes comparing both of these groupings, as illustrated in Additional file 4: Figure S4D. The mtPCDI prognosis model and heatmap of the 18 mtPCDI signature genes are presented in Fig. 3A. The TSC/mTOR, RTK, PI3K/AKT, RAS/MAPK, Hormone ER, DNA Damage Response, Hormone AR, EMT, Cell Cycle, and Apoptosis pathways are widely recognized as being closely linked to cancer. To evaluate the activity levels, we classified the samples into a pair of categories (High and Low) on the basis of a median gene expression. To determine the effect of a gene on a pathway, we used pathway activity score (PAS) (gene low expression) < PAS (gene high expression) to indicate activation and the opposite for inhibition (Fig. 3B). Figure 3C provides an overview of the relationship between the 18 mtPCDI hallmark genes expression and the 24 immunological kinds of cell infiltrated in LGG. Additionally, an analysis was performed to identify potential pathways associated with each risk gene. As illustrated in Fig. 3D and E, a total of 186 pathways were significantly correlated with these 18 genes, encompassing Cell cycle, biological metabolism, and immune activity, among others. We then delved into the role of the 18 mtPCDI signature genes’ expression on immune subtypes (Additional file 5: Figure S5) and molecular subtypes (Additional file 6: Figure S6) among LGG using the TISIDB website.

Based on our findings, we conclude that the 18 signature genes exhibit differential expression in various immunological and molecular subtypes of LGG.

### Multi-omic comparison between two mtPCDI groups

**Fig. 4:**
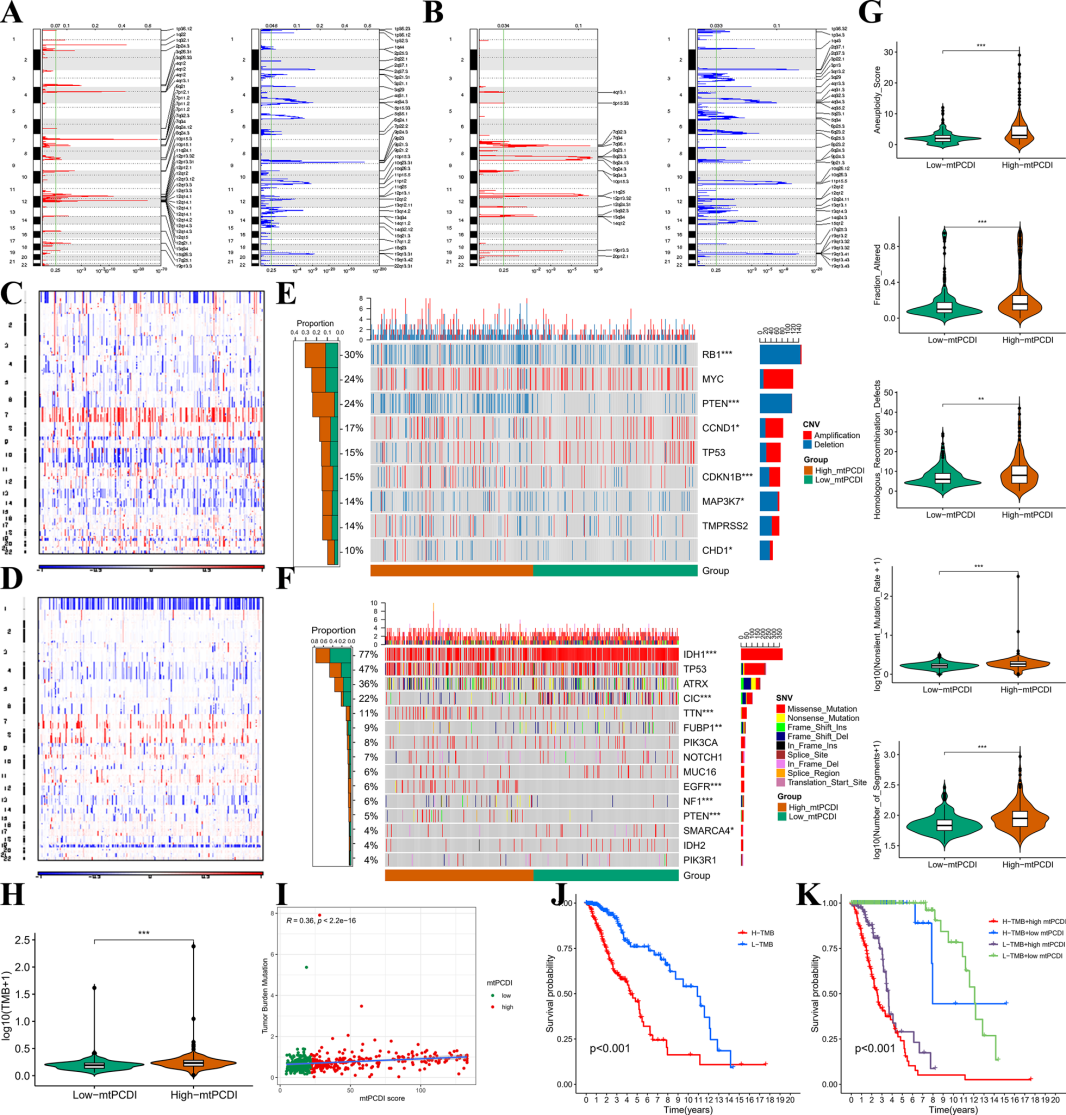
Multi-omics characterisation. Recurrent regions of copy number amplification and deletion in the (**A**) mtPCDI-high and (**B**) low-mtPCDI. Different profiles of copy number alterations observed between LGG patients with high- and low-mtPCDI. **C**–**D**, landscape with 1p/19q co deletion incidence in high and low mtPCDI subset. **E** Oncoprint showing genes affected by recurrent copy number alterations, with corresponding proportions of alterations in each group depicted in the bar plot on the right. **F** Oncoprint showing common somatic gene mutations, with corresponding proportions of mutations in each group depicted in the bar plot on the right. **G** Comparison of aneuploidy score, fraction altered, homologous recombination defects, nonsilent mutation rate, and number of segments between high- and low-mtPCDI patients in the TCGA-LGG dataset. **H** Comparison of high- and low-mtPCDI subgroups of TMB. **I** Mutation load and mtPCDI correlation analysis. **J** Kaplan–Meier curve of OS for patients classified by mtPCDI. **K** mtPCDI and TMB-categorized OS Kaplan–Meier curves. *p < 0.05; **p < 0.01; ***p < 0.001; ns, no statistical significance

Using GISTIC2.0, we identified that the mtPCDI-high group displayed a higher frequency of recurrent copy number alterations compared to the low-mtPCDI group (Fig. 4A, B). We then utilized CNA information to investigate specific chromosomal modifications. Notably, we observed a higher frequency of Chr 7 amplification coupled with Chr 10 loss, a major characteristic of glioma [53], in the high-mtPCDI subset (Fig. 4C). Conversely, the low-mtPCDI group had a higher occurrence of the 1p/19q codeletion, a genomic indicator of oligodendroglioma (Fig. 4D) [54]. Furthermore, an analysis of genes impacted by somatic copy number alterations unveiled a heightened occurrence of deletions in tumor suppressor genes, such as RB1, PTEN, and MAP3K7, among different LGG patients (Fig. 4E; all p < 0.05). We observed frequent amplification of CCND1, CDKN1B, and CHD1 in the high-mtPCDI group (all p < 0.05). Additionally, we compared common somatic mutations in individuals with high and low mtPCDI (Fig. 4F) and discovered significant IDH1 mutations were more frequent in low-mtPCDI patients compared those with high-mtPCDI patients. Moreover, the high-mtPCDI group exhibited significantly elevated aneuploidy score, proportion changed, homologous recombination faults, nonsilent mutation rate, number of segments, and tumor mutation load (TMB) in comparison to the low-mtPCDI group (all p < 0.05, Fig. 4G, H).

To investigate the correlation between TMB and mtPCDI, we found that TMB was noticeably more abundant in the high-mtPCDI subgroup (p < 0.001; Fig. 4H). TMB and mtPCDI also showed a moderately positive correlation (R = 0.36, p < 0.001; Fig. 4I). It is noteworthy that patients with lower TMB scores had better survival outcomes compared to those with higher TMB scores (Fig. 4J). To unravel the synergistic or antagonistic potential of TMB and mtPCDI in predicting survival, we categorized patients based on these factors and conducted survival analysis. Patients with low TMB and mtPCDI had the most favorable prognoses, while those with high TMB and mtPCDI had the worst outcomes (Fig. 4K).

These findings suggest a potential association between mutational burden and response to immunotherapy, providing a novel perspective on checkpoint blockade treatment. Overall, the genomic pattern of the mtPCDI-high group resembled that of advanced LGG.

### The underlying biological mechanisms of mtPCDI groups

**Fig. 5:**
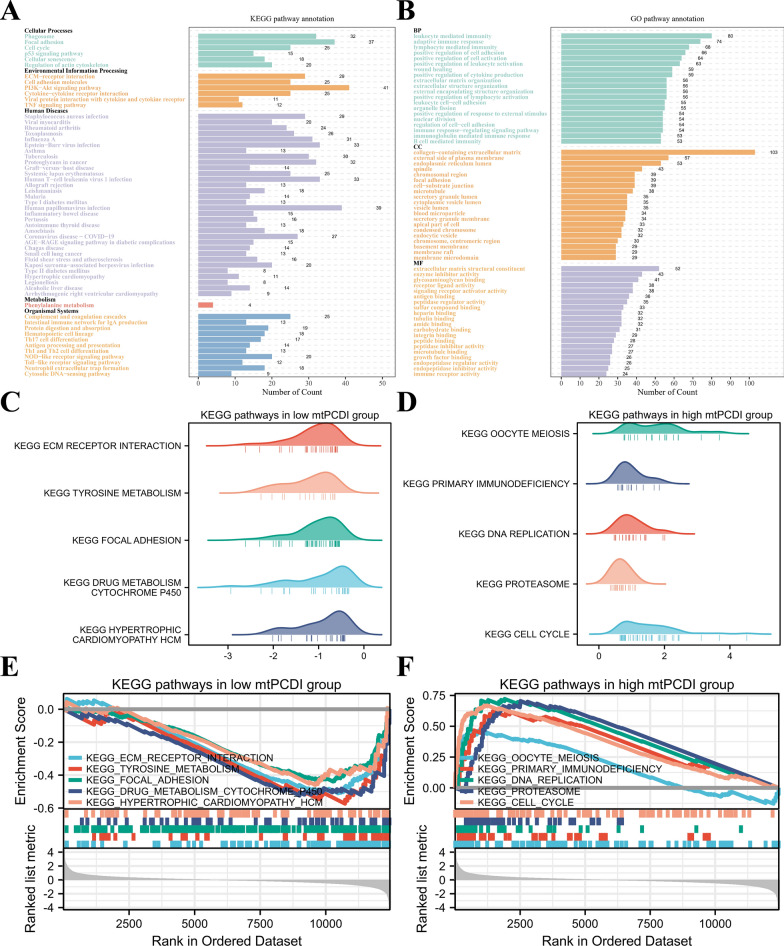
Functional enrichment analysis of mtPCDI groups. **A** KEGG and **B** GO enrichment analyses of mtPCDI groups. The top 5 GSEA enriched pathways in the **C**, **E** low-mtPCDI **D**, **F** and high-mtPCDI groups

To delve deeper into the biological processes linked to mtPCDI groups, we executed enrichment analysis. The Kyoto Encyclopedia of Genes and Genome (KEGG) results are presented in Fig. 5A. Regarding cellular processes, mtPCDI were predominantly enriched in focal adhesion, phagosome, and cell cycle. In terms of environmental information processing, mtPCDI appeared most highly concentrated in the PI3K-Akt signaling pathway, ECM-receptor communication, and cytokine-cytokine receptors connection. Concerning organismal systems, mtPCDI were particularly abundant in cytosolic DNA-sensing route, complementing and anticoagulant chain reactions, and the NOD-like receptor signaling pathways. The Gene ontology (GO) results are presented in Fig. 5B. Concerning biological processes (BP), the genes associated with mtPCDI were primarily enriched in leukocyte-mediated protection, lymphocyte-mediated the body’s defense, and immune response adaptation. Regarding cellular components (CC), the genes associated with mtPCDI were primarily rich in a matrix of cells that contains collagen, the endoplasmic reticulum (ER) lumen, and plasma membrane’s outer layer. The primary molecular functions (MF) of mtPCDI were concentrated in the matrix of cells structural component, glycosaminoglycan enforceable, and enzyme inhibition activity.

In addition, we utilized GSEA to identify potential pathways associated with mtPCDI. As depicted in Fig. 5C and E, the low-mtPCDI group was significantly enriched for environmental information processing and cellular process-related pathways, such as ECM receptor interaction and focal adhesion. In contrast, the high-mtPCDI group was predominantly associated with DNA replication, cell cycle, and other biological processes related to proliferation, which partially explained its more advanced grades and unfavorable prognosis, as depicted in Fig. 5D and F. The supplementary distinctions in biological pathways between the two mtPCDI groups are presented in Additional file 7: Figure S7.

### Potential biological mechanisms related to the mtPCDI signature

**Fig. 6:**
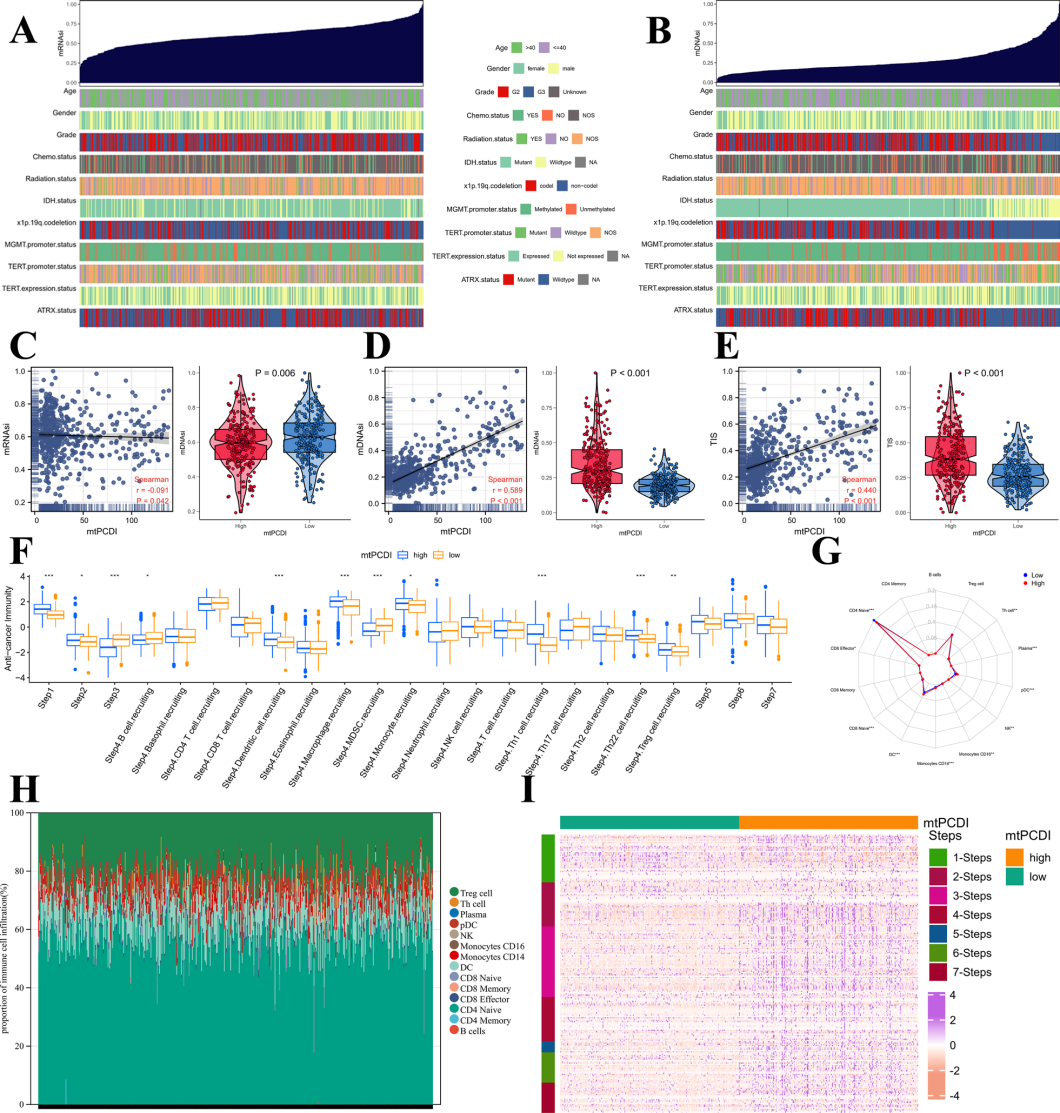
Functional annotation of the mtPCDI signature. **A** Association between known clinical and molecular features (age, gender, grade, chemotherapy status, radiotherapy status, IDH1 status, MGMT promoter methylation status, 1p/19q codeletion, TERT promoter methylation status, TERT expression status, and ATRT status) and mRNAsi in LGG. **B** Association between known clinical and molecular features (age, gender, grade, chemotherapy status, radiotherapy status, IDH1 status, MGMT promoter methylation status, 1p/19q codeletion, TERT promoter methylation status, TERT expression status, and ATRT status) and mDNAsi in LGG. **C** Correlation between mRNAsi and mtPCDI, and mRNAsi difference between two mtPCDI groups. **D** Correlation between mDNAsi and mtPCDI, and mDNAsi difference between two mtPCDI groups. **E** Correlation between TIS and mtPCDI, and TIS difference between high- and low-mtPCDI groups. **F** Box plot portrays the dissimilarities in cancer immunity cycle between two mtPCDI groups. **G** Radar plot displaying the tumor-infiltrating immunecells between two mtPCDI groups. **H** The bar plot shows the relative proportion of tumor-infiltrating immunecells. **I** Heatmap illustrates the expression levels of 178 step-specific signature genes associated with anti-cancer immunity across all samples in the seven-step Cancer-Immunity Cycle. *p < 0.05; **p < 0.01; ***p < 0.001; ns, no statistical significance

Cancer stem cells (CSCs), are cancerous cells that exhibit traits associated with normal stem cells and have the ability to produce every type of cell observed in a particular cancer sample. To investigate the gene expression and epigenetic profiles of CSCs, we calculated mRNAsi scores and mDNAsi scores, respectively, in TCGA-LGG samples. Heatmaps of mRNAsi and mDNAsi scores against clinicopathological information are presented in Fig. 6A and B, while the correlation and variability of mRNAsi and mDNAsi scores with mtPCDI are displayed in Fig. 6C and D. Specifically, higher mtPCDI values were associated with lower mRNAsi scores and higher mDNAsi scores. Furthermore, higher mtPCDI characteristics were linked to a higher TIS, as shown in Fig. 6E.

To investigate potential biological mechanisms associated with the mtPCDI signature, and the progression of cancer protection was estimated. Interestingly, in known as high mtPCDI team, four phases in the tumors immune process had been additional engaged, which includes phase 1 (antigen release), phase 2 (cancer antigen presentation), phase 3 (priming and activation), and phase 4 (tumor immunized infiltrating cells recruitment). However, three phases in the cancers immunized period were inert, including step 5 (immune tissues influx), phase 6 (cancer cells comprehension by T cells), and phase 7 (cancer cells executing) in the TCGA-LGG, as shown in the Fig. 6F. Additionally, we calculated the tumour-infiltrating immune cell scores to explore potential immunological mechanisms associated with the mtPCDI profile. As depicted in Fig. 6G and H, high mtPCDI were broadly associated with higher levels of CD4 Naive, CD8 Effector, CD8 Naive, DC, Monocytes CD14, Monocytes CD16, NK, pDC, Plasma, and Th cells in the TCGA LGG developed for tumour characteristics. Furthermore, we investigated the expressing themselves quantities of 178 step-specific that it has been signed genes associated with cancer-fighting protection in the seven-phase Cancer-Immunity process across really samples. The results of this analysis are displayed in Fig. 6I.

### Immune characteristics

**Fig. 7:**
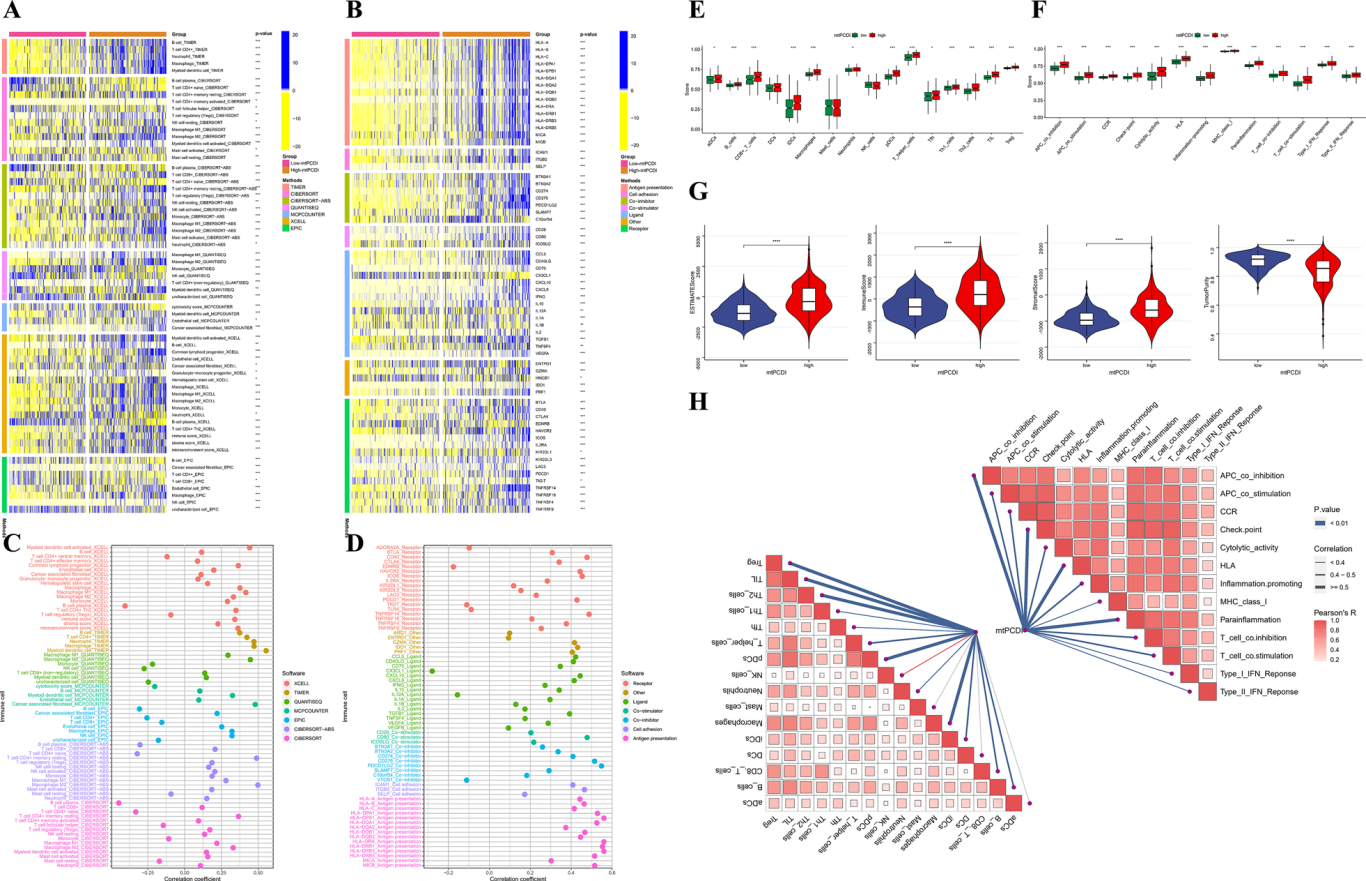
Exploration of the tumour immune microenvironment. **A** Immune infiltrating cells were estimated by using multiple algorithms between the two mtPCDI subgroups. **B** Immune modulator molecules were estimated between the two mtPCDI subgroups. **C** Correlation analysis to estimate the number of immune-infiltrating cells. **D** Correlation analysis to estimate the presence of immune modulator chemicals. Differences in scores for infiltrating immune cells **E** and immune-related functions **F** between two mtPCDI groups. **G** Estimatescore, immunescore, stromalscore, and Tumorpurity in the two mtPCDI groups. **H** butterfly plot to illustrate the correlation between mtPCDI and infiltrating immune cells, as well as immune-related functions. *p < 0.05; **p < 0.01; ***p < 0.001; ns, no statistical significance

To assess the role of mtPCDI markers in LGG in the tumor immune microenvironment, we investigated the relationship between mtPCDI grouping and mtPCDI markers with immune cell infiltration and immunomodulators, respectively. Our analysis revealed that the high-mtPCDI group exhibited more immune cell infiltration based on seven immune infiltration algorithms (Fig. 7A). To explore the differences in immunomodulator expression between the two subgroups, we examined the expression of immunomodulators (antigen presentation, cell adhesion, co-inhibitors, co-stimulators, ligands, receptors, etc.), showing higher expression in the high-mtPCDI group (Fig. 7B). Furthermore, the mtPCDI feature score was constructively associated with nearly all malignancy immune contaminating tissue cells, such as Myeloid dendritic cells, Macrophage M1, T cells CD4 + , Neutrophil, Macrophage, and Myeloid dendritic cells in TCGA-LGG, and Macrophage M2 (Fig. 7C). Additionally, mtPCDI characteristic scores were positively correlated with most immune system modulators, especially PDCD-1, CTLA-4, and IDO-1 (Fig. 7D). Additionally, we used the ssGSEA enrichment score to explore the relationship between mtPCDI and distinct immune cell subpopulations and activities. Our results showed that, with the exception of mast cells and NK cells, ssGSEA scores were significantly greater in high-mtPCDI patients for practically all immune-related cell functions (Fig. 7E). Similarly, we observed in the high-mtPCDI sample compared to the low-mtPCDI group, there was a greater enhancement of inflammatory conditions, checkpoint, cytolytic exertion, T cell co-inhibition, T cell co-stimulation, parainflammation, and type 2-related IFN reactions, higher immune function scores for major histocompatibility complex (MHC) class I, APC co-inhibition, chemokine receptor (CCR), and human leukocyte antigen (HLA) (Fig. 7F). The StromalScore, ImmuneScore, and ESTIMATEScore were considerably lower in the patients with high mtPCDI (all p < 0.001) than those in the low-mtPCDI group, as shown by the tumor microenvironment scores in Fig. 7G. Lastly, the association between mtPCDI and various immune cell subsets and functions is depicted in Fig. 7H, demonstrating increased immunological activity in the group with elevated mtPCDI.

### Establishment of a nomogram

**Fig. 8:**
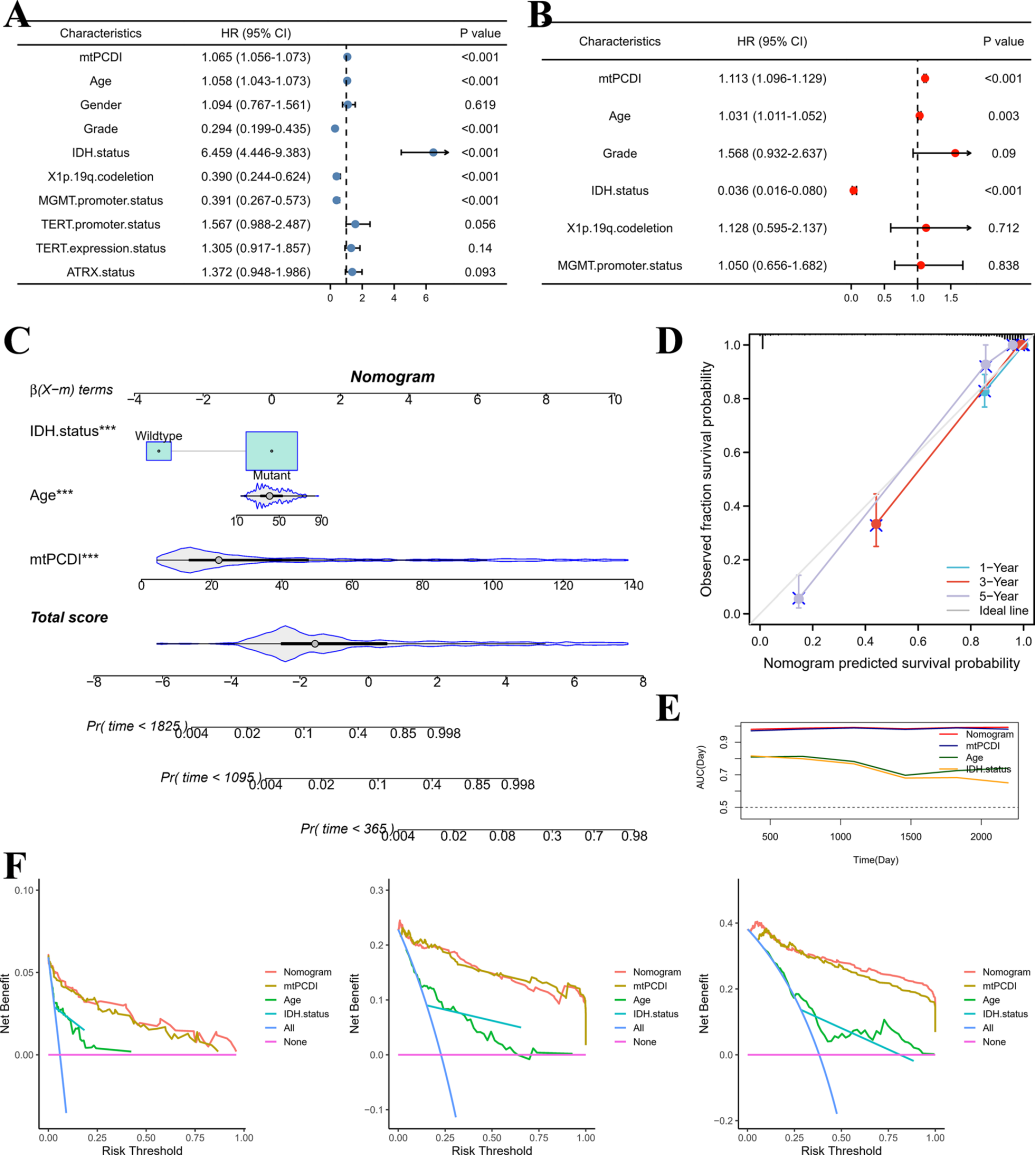
Building and validating nomograms. **A** Univariate and **B** Multifactorial analyses. **C** Nomogram to predict 1-, 3-, and 5-year survival. **D** Nomogram calibration curves for 1-, 3-, and 5-year OS. **E** AUC analysis of variables included in the nomogram model. **F** DCA curves were compared over a period of 1 year, 3 years, and 5 years for patients with LGG

We created and assessed a nomogram structure built around mtPCDI using multivariate as well as univariate cox regression in order to better examine the prediction usefulness of mtPCDI. According to our research, mtPCDI is a standalone predictive factor for those having LGG (Fig. 8A, B). We then constructed a nomogram model using multivariate Cox regression to estimate OS at 1-, 3-, and 5-years. The model included age, IDH1 status, and mtPCDI, as displayed in Fig. 8C. Figure 8D shows the model's measurement curves, which show that the nomination accurately predicted the 1-, 3-, and 5-year mortality rates for LGG sufferers. Additionally, the AUC analysis of the model demonstrated a high diagnostic value for this nomogram plot, as depicted in Fig. 8E. As illustrated in Fig. 8F, the nomogram model demonstrated significant net benefits across a broad spectrum of risks, according to the DCA findings. Overall, our results suggest that the nomogram model based on mtPCDI has a strong performance in predicting the prognosis of LGG patients.

### Analyses of chemotherapy drug sensitivity

Our analysis revealed 13 chemotherapeutic agents for the treatment of LGG based on the results of oncoPredict, including Lisitinib, LFM.A13, and STF.62247, among others (Additional file 8: Figure S8A, Additional file 16: Table S8). Similarly, based on the results of pRRophetic, we identified 16 chemotherapeutic agents for LGG, such as Crizotinib, Sorafenib, Saracatinib, and Paclitaxel, among others (Additional file 8: Figure S8B, Additional file 17: Table S9). The high sensitivity of these drugs in LGG patients indicates that such chemotherapeutic agents may be beneficial for individuals afflicted with this condition.

### Consensus clusters identified two clusters of LGG patients

**Fig. 9:**
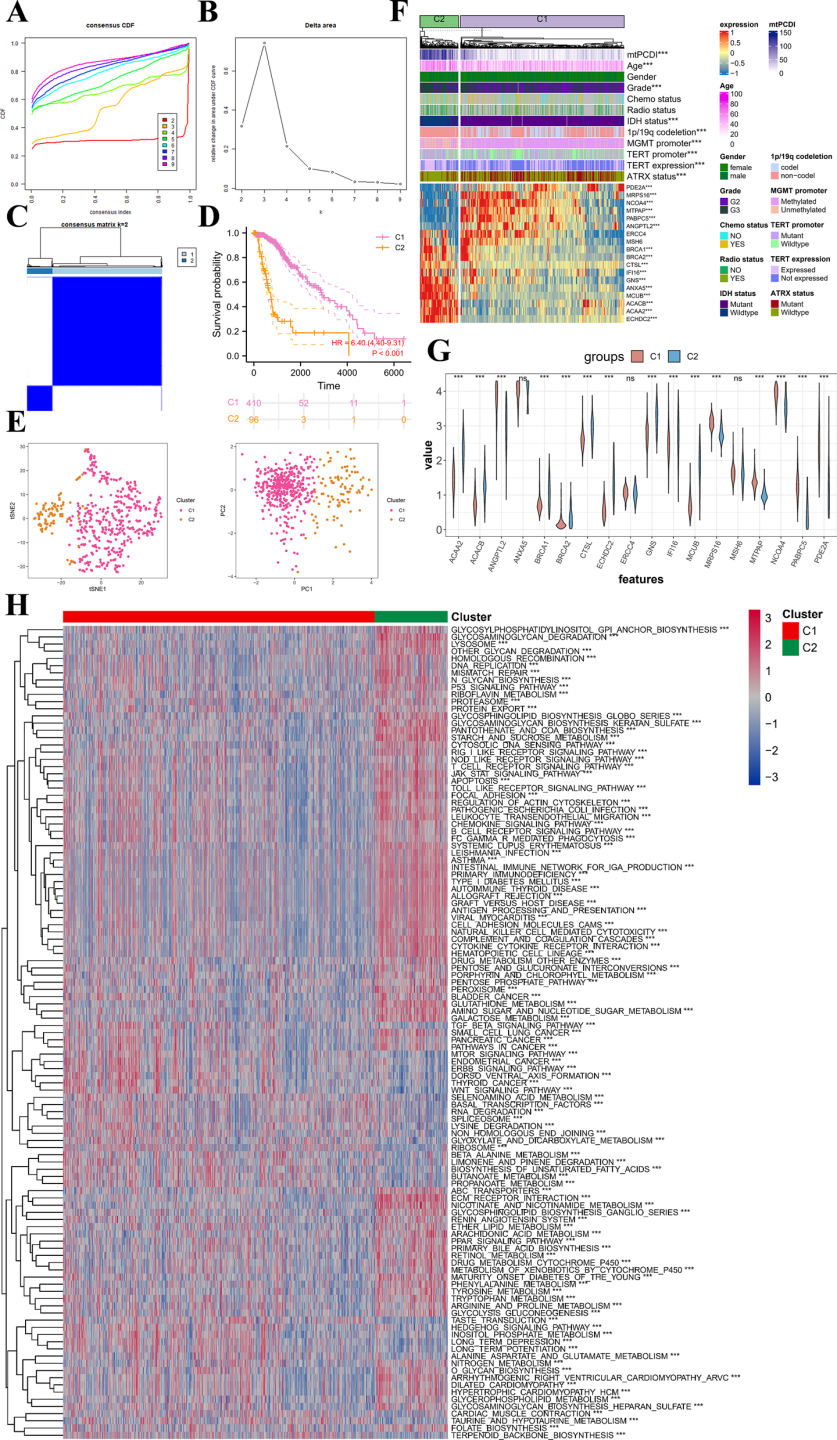
Two different LGG clusters were delineated by consensus clustering. **A**, **B** Consensus clustering CDF for k = 2 to 9. **C** The consensus clustering with k = 2. **D** Kaplan–Meier survival curves of LGG patients’ OS among the two subgroups. **E** tSNE analysis and PCA analysis between two different subgroups. **F** Heatmap showing the between two different subgroups and clinical features and expression of 18 most valuable genes in LGG patients. **G** Analysis of differences in the expression of mtPCDI signature genes in two different subgroups. **H** Pathway activity between two different subgroups based on ssGSEA algorithm. *p < 0.05; **p < 0.01; ***p < 0.001; ns, no statistical significance

To explore the involvement of mtPCDI signature genes in LGG development, we performed a consistent clustering analysis of the activity values of the 18 signature markers. For the most stable clustering, K = 2 was deemed appropriate (Fig. 9A, B). This analysis resulted in the division of all LGG samples into two subgroups: C1 (n = 410) and C2 (n = 96) (Fig. 9C). Importantly, there was plenty of variations in OS among the two subgroups (p < 0.001, Fig. 9D). C1 was predominantly composed of low-mtPCDI patients, while Cluster 2 was mainly composed of high-mtPCDI patients. The t-SNE and PCA analyses (Fig. 9E) further revealed dimensional variations among the two groupings were considerable. Additionally, the heatmap of mtPCDI Consensus clusters and clinical pathological variables (Fig. 9F) demonstrated that, apart from gender, radiotherapy, and chemotherapy status, the rest of the clinicopathological characteristics were substantially different compring the C1 and C2 sections (all p < 0.001). Furthermore, the pathway-based ssGSEA results (Fig. 9H) indicated that the C2 subtype activated more tumour and immune-related pathways, such as DNA replicating, homologous recombination (HR), P53 signaling pathway, mismatch repair, T cell receptor signaling pathway, JAK STAT signaling pathway, B cell receptor signaling pathway, and apoptosis, suggesting that the mtPCDI signature is closely associated with these typical tumour-related pathways.

### Cluster-based analysis of tumor immune microenvironments

**Fig. 10:**
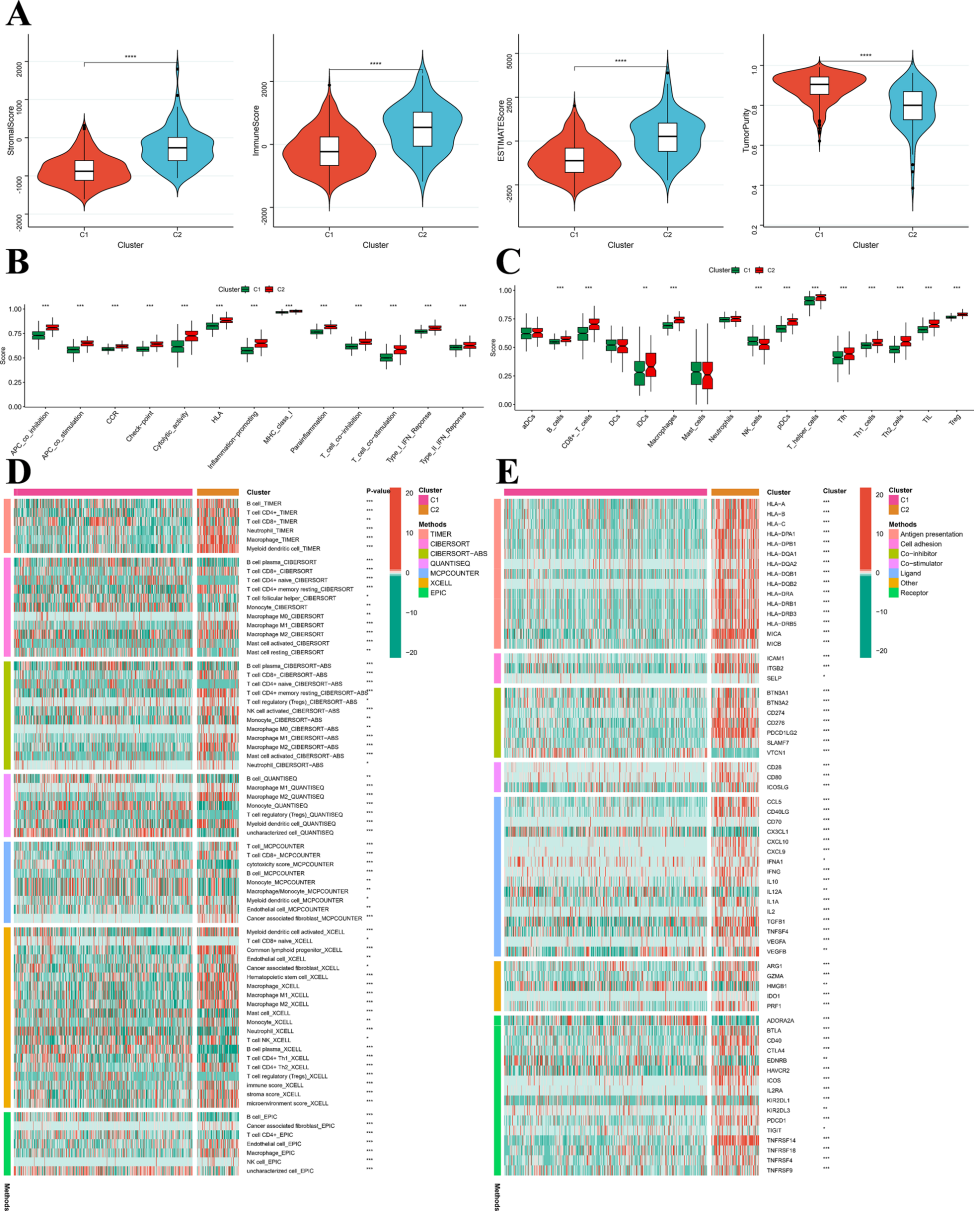
Exploration of the immune status of tumours between the two different subgroups. **A** Estimatescore, immunescore, stromalscore, and Tumorpurity in the C1 and C2 subgroups. The score of the **B** infiltrating immune cells and **C** immune-related functions in the C1 and C2 subgroups. **D** Immune infiltrating cells were estimated by using multiple algorithms between the two different subgroups. **E** Immune modulator molecules were estimated between the two different subgroups. *p < 0.05; **p < 0.01; ***p < 0.001; ns, no statistical significance

Our results suggest that individuals with the C2 subtype had higher levels of immune score, stromal score, and estimate score, while having lower levels of tumor purity (Fig. 10A). Moreover, using ssGSEA enrichment scores, we assessed the relationship between consensus clusters and various immune cell subsets and functions. The results showed that nearly all immune function scores were higher in the C2 subtypes than in the C1 ones (Fig. 10B), almost all functional immune cells exhibited significantly higher ssGSEA scores in patients with the C2 subtype, except for DCs and Mast cells (Fig. 10C). Additionally, the C2 subtype showed a stronger ImmuneScore, suggesting that it may mount a more robust response to immunotherapy (Fig. 10D). Based on our findings, we concluded that the C2 subtype serves as a representative of the immune system and displays higher expression of immunomodulators such as antigen presentation, cell adhesion, co-inhibitors, co-stimulators, ligands, receptors, among others, compared to the C1 cluster (Fig. 10E). Therefore, the C2 subtype exhibits greater immune activity.

### Pan-cancer analysis of mtPCDI signature genes

**Fig. 11:**
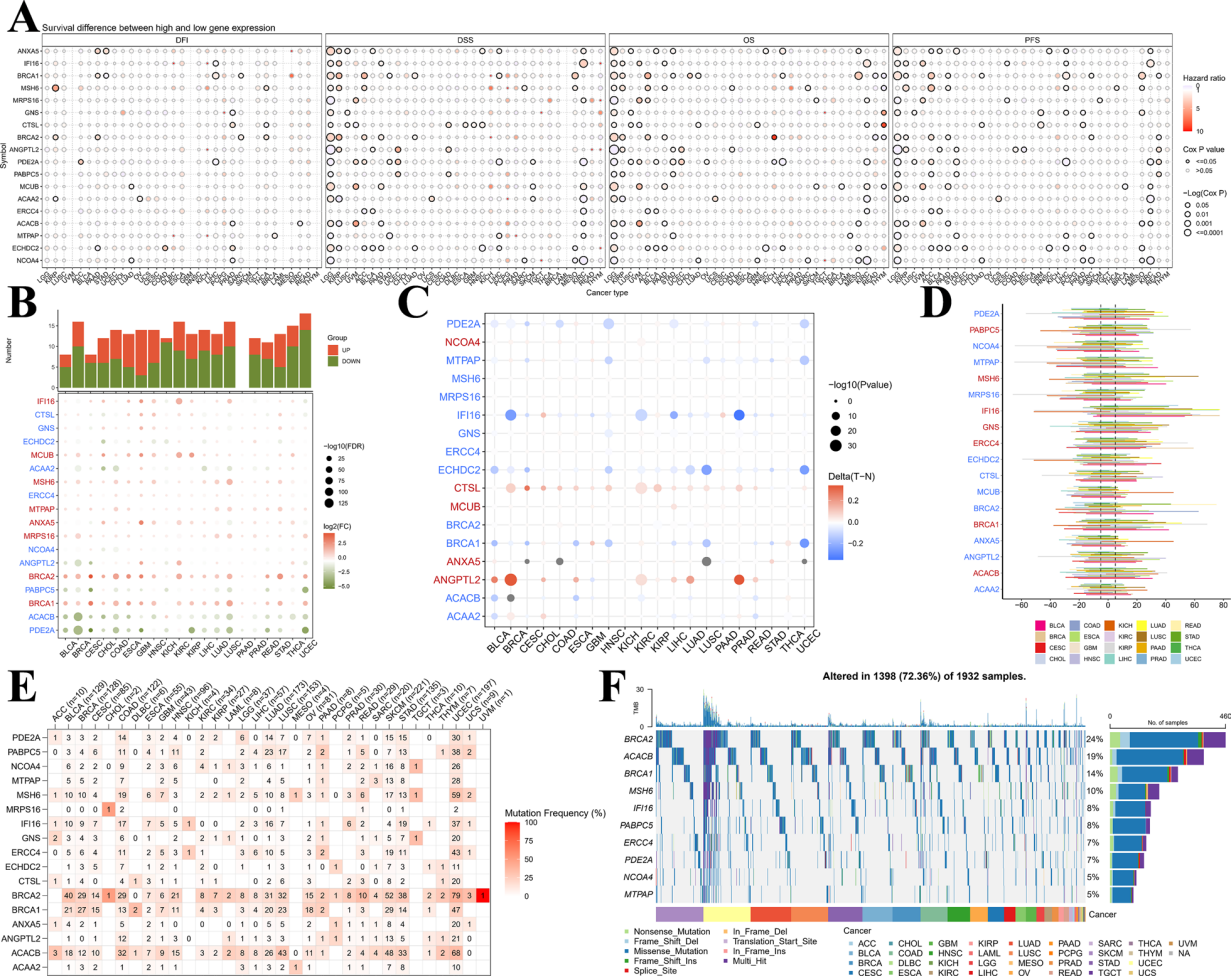
The landscape of mtPCDI signature genes in pan-cancer analysis. **A** Presents hazard ratios and Cox P values for selected cancer types and genomic symbols. Rows represent genomic symbols, while columns denote cancer types. The color and size of the bubbles indicate hazard ratios and significance of Cox P values, respectively. A blue to red color gradient is used to represent low to high hazard ratios, and larger bubbles correspond to greater statistical significance of Cox P values. The black outline border highlights the Cox P value ≤ 0.05 threshold. In **B**, graphs exhibit logFC and FDR of signature genes for each cancer type. Red and blue colors indicate clearly up- and down-regulated genes, respectively. Additionally, **C** shows a heatmap demonstrating varied methylation patterns of signature genes in malignant tumors. Hypermethylated and hypomethylated genes are represented in red and blue, respectively, employing Wilcoxon rank-sum test the degree of statistical significance assessed. **D** is a bar graph that presents frequency of copy number variation changes for each signature gene and cancer type. Finally, **E** and **F** demonstrate signature gene mutation frequency and single-nucleotide variant oncoplot for a given malignancy, respectively

Our study repeatedly confirmed the important value of the 18 mtPCDI signature genes in LGG as described above, but to summarize the pan-cancer spectrum of these 18 mtPCDI signature genes, an in-depth investigation into the involvement of these genes across various human malignancies, encompassing expression profiles, predictive capacity, methylation patterns, CNV, and SNV, is of paramount importance. To begin, we developed survival profiles for the concerned genes by leveraging the connection between gene expression levels, as documented by TCGA, and patient survival outcomes (Fig. 11A). We then searched the TCGA database to compare the gene expression of tumour tissues with that of healthy samples. We found that the expression of IFI16, MCUB, MSH6, MTPAP, ANXA5, MRPS16, BRCA2 and BRCA1 genes were usually upregulated in cancer tissue, while the expression of the remaining genes was usually downregulated (Fig. 11B). Altered SNV, CNV and methylation of these five signature genes, including LGG, were evident. In most cancers, the signature genes were differentially methylated compared to normal tissue; in particular, genes including as representatives NCOA4, CTSL, MCUB, ANXA5 and ANGPTL2 were usually hypermethylated, while genes including PDE2A and others were usually hypomethylated (Fig. 11C). Substantial CNV deletions were observed in PABPC5, MSH6, IFI16, GNS, ERCC4, BRCA1 and ACACB, while substantial CNV amplification was observed in most tumour types for the remaining genes (Fig. 11D). Significant SNV alterations were observed in BRCA2, ACACB, BRCA1, MSH6 and IFI16 in most tumour types (Fig. 11E, F).

### Immunohistochemistry of mtPCDI signature genes

**Fig. 12:**
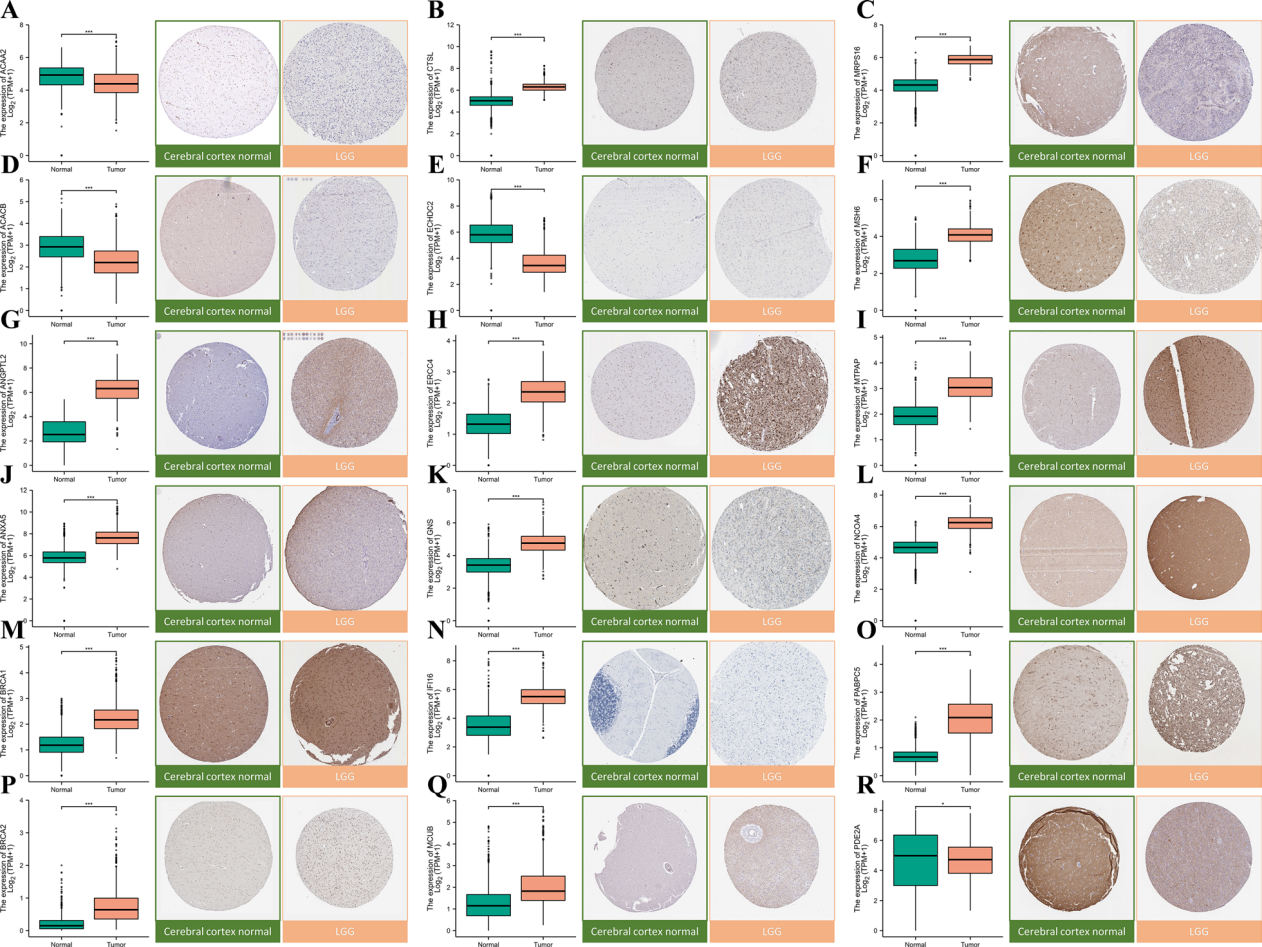
Gene expression and immunohistochemical analysis of 18 mtPCDI signature genes. **A**–**R** show results for ACAA2, CTSL, MRPS16, ACACB, ECHDC2, MSH6, ANGPTL2, ERCC4, MTPAP, ANXA5, GNS, NCOA4, BRCA1, IFI16, PABPC5, BRCA2, MCUB, and PDE2A. *p < 0.05; **p < 0.01; ***p < 0.001; ns, no statistical significance

We collected IHC staining images of 18 mtPCDI signature genes-associated proteins from the HPA database, which were obtained from both LGG and healthy brain tissue. Our primary objective was to explore any possible differences in protein expression levels between these two sample types for the mentioned 18 genes. Significantly higher protein expression levels were observed for 18 of the mtPCDI signature genes in LGG samples relative to normal samples, thereby supporting our findings (Fig. 12A–R).

## Discussion

LGG is a complex and heterogeneous disease, and predicting the prognosis of LGG patients is challenging. Prognostic factors play a crucial role in predicting disease progression, selecting appropriate treatment strategies, and estimating overall survival [55]. Among the significant prognostic factors, histological subtype and grade hold paramount importance. Higher grades are associated with more aggressive tumor behavior, shorter progression-free survival, and reduced overall survival. Molecular markers have emerged as powerful predictors of prognosis in LGG. Age at diagnosis is another crucial factor influencing prognosis, with younger patients tending to exhibit longer overall survival than older patients. Tumor extent and location carry prognostic implications, with smaller tumor size and accessible locations associated with better outcomes. Residual disease after surgical resection is another important factor influencing prognosis. Other prognostic factors include O^6-methylguanine-DNA methyltransferase promoter methylation status, genetic alterations beyond IDH and 1p/19q, and the impact of adjuvant therapy. As our understanding of LGG biology continues to expand, emerging prognostic factors and novel molecular markers are being investigated to refine prognostication and personalize treatment approaches.

Machine learning techniques are becoming more prevalent in predicting patient survival. Nevertheless, effectively implementing these methods in clinical practice while maintaining accuracy remains a challenge. However, two questions worth considering are why particular machine learning algorithms should be used and which solution is the best one. A researcher's choice of algorithm may depend heavily on their own preferences and biases [56]. In this research, we gathered expression files from 1467 LGG patients from six multicentre cohorts worldwide and used a novel computational framework to explore the crosstalk between mitochondrial function and 18 cell death patterns. The mtPCDI was based on the expression of 18 genes (ACAA2, ACACB, ANGPTL2, ANXA5, BRCA1, BRCA2, CTSL, ECHDC2, ERCC4, GNS, IFI16, MCUB, MRPS16, MSH6, MTPAP, NCOA4, PABPC5, and PDE2A) that were most potent in predicting patient survival. Our study findings indicate that mtPCDI can serve as a valuable tool in guiding treatment decisions and improving patient outcomes. By identifying these genetic alterations through mtPCDI, clinicians can gain important insights into the underlying molecular mechanisms driving the tumor growth. This information can help guide the selection of targeted therapies or treatment strategies that specifically address the identified genetic abnormalities.

ANGPTL2 is a secreted protein that has been demonstrated to facilitate tumor growth and invasion in various cancer types, including glioma. Both glioma tissues and cells have shown significant elevations of ANGPTL2 expression levels. Nevertheless, ANGPTL2 knockdown induced a significant decline in the invasive capacity and proliferation of glioma cells. Additionally, tumorigenesis assays demonstrated that inhibiting ANGPTL2 caused a decrease in glioma tumor growth in vivo. Noteworthy, ANGPTL2 silencing led to a reduction in the protein levels of p-ERK1/2 in glioma cells, consequently impeding the ERK/MAPK signaling pathway’s activity [57]. These findings indicate that ANGPTL2 might have a critical role in glioma’s emergence and progression, and targeting this protein could be a potential therapeutic approach for glioma treatment. The ANXA5 gene, which is situated on 4q27 in humans, encodes for Ca2 + -regulated phospholipid- and membrane-binding proteins. Increasing evidence suggests that ANXA5 may be involved in carcinogenesis by inhibiting the activity of protein kinase C in the RTK-Ras/Raf/MEK/ERK signaling pathway [58, 59]. Moreover, ANXA5 has a broad distribution and has been observed to exhibit abnormal expression in various cancer types, such as prostate cancer, cervical carcinoma, and cholangiocarcinoma [60, 61]. According to research, non-angiogenic gliomas are characterized by express higher levels of ANXA5 (with a 2.1-fold increase) and angiogenic glioma (with a 3.4-fold increase) as compared to normal tissues [62]. In LGG, mutations in BRCA1 and BRCA2 have been associated with a higher risk of tumor recurrence and a poorer prognosis. CTSL is a lysosomal protease that has been implicated in the regulation of apoptosis and autophagy. In LGG, methionine deprivation to downregulate CTSL and induce proliferation inhibition in glioma cells [63]. IFI16 has been found to inhibit apoptosis and enhance cell survival by activating the NF-κB pathway. MSH6 is a DNA mismatch repair protein that is involved in the maintenance of genome stability. LGG has been linked to a greater risk of tumor recurrence and a poorer prognosis resulting from mutations in MSH6. NCOA4, a nuclear receptor coactivator, is involved in the regulation of autophagy and iron metabolism. Moreover, NCOA4 has been discovered to induce apoptosis by activating the caspase-3 pathway. On the other hand, PABPC5, a poly(A)-binding protein, has been implicated in regulating mRNA stability and translation. It has also been found to exert an inhibitory effect on apoptosis while to promote cell survival by activating the PI3K/Akt pathway. The organelles called mitochondria play a crucial role in ATP production and the regulation of cellular metabolism. There is a suggestion that dysfunctional mitochondria play a notable role in regulating cell survival, metabolism, and proliferation in cancer. In LGG, the regulation of mitochondrial function has been linked to several genes including ACAA2, ACACB, ECHDC2, MCUB, MRPS16, MTPAP, and PDE2A. ACAA2 is a key enzyme involved in the beta oxidation of lipid acids. It works closely with its isoenzyme, ACAA1, to catalyze the beta oxidation process through a complex mechanism [64]. Inhibiting ACACB has demonstrated a decrease in the proliferation and de novo lipogenesis of EGFRvIII human glioblastoma cells [65]. MCUB is involved in facilitating glioma invasion/migration under hypoxic conditions [66]. The activation of the PI3K/AKT signaling pathway by MRPS16 leads to elevated levels of Snail protein expression, thereby promoting glioma progression [67]. The main transcript of PDE2A/miR-139 has been recognized as a crucial factor in hindering the Wnt/β-catenin pathway, which eventually results in the regulation of GSC stemness and the development of tumors [68]. In conclusion, the regulation of programmed cell death and mitochondrial function plays a critical role in the development and progression of LGG.

Our investigation revealed the presence of shared genes impacted by somatic copy number alterations and significant disparities in somatic mutations between the mtPCDI-high and low-mtPCDI cohorts. Interestingly, the high mtPCDI group demonstrated significantly higher TMB, which has emerged as a novel prognostic biomarker closely associated with the response to immunotherapy. We established a moderately positive correlation between TMB and mtPCDI and discovered a potential link between mutational burden and immunotherapy response, providing a new perspective on checkpoint blockade treatment. Moreover, our analysis revealed that the high-mtPCDI group demonstrated increased immune cell infiltration and higher expression levels of immunomodulators, including classical immune checkpoint molecules. However, patients classified in the high-mtPCDI group exhibited notably lower levels of StromalScore, ImmuneScore, and ESTIMATEScore compared to those in the low-mtPCDI group. This suggests a complex interplay between the mtPCDI and the tumor immune microenvironment. Overall, our findings suggest that mtPCDI may serve as a valuable biomarker for predicting the genomic pattern and response to immunotherapy in LGG patients.

As a result of our enrichment analysis, we found that mtPCDI-related genes were mainly enriched in various cellular processes, environmental information processing, and organismal systems. Of particular interest, our analysis uncovered a significant correlation between the high mtPCDI group and DNA replication, cell cycle, and other proliferation-related biological processes. These findings provide partial insight into the more unfavorable prognosis observed in this group.

One notable limitation of this study is the lack of in vitro or in vivo experiments to directly validate our findings. While we have extensively utilized bioinformatics analyses and computational methodologies, experimental validation remains an essential aspect of scientific research. In experiments could provide valuable insights into the functional implications of the observed patterns and strengthen the credibility of our results. Therefore, future research should focus on conducting targeted experiments that can corroborate and extend the observations from mtPCDI.

## Conclusion

In conclusion, our findings have enabled us to construct a mtPCDI signature within the TCGA cohort, which we have further validated across five other external cohorts, demonstrating superior performance. Of particular importance, our mtPCDI maintains its status as the most potent prognostic indicator even following adjustment for potential confounding factors, surpassing established clinical models in predictive strength. Additionally, our investigation into the association between mtPCDI and immunomodulators, tumor microenvironment, and drug sensitivity provides valuable insights for future in-depth studies.

### Supplementary Information

**Additional file 1: Figure S1.** Kaplan–Meier mortality line was calculated of OS between patients with high or low expression of 18 most valuable.

**Additional file 2: Figure S2.** Differential analysis of mtPCDI correlation between clinical and pathological variables among different subgroups. Age, gender, tumor levels, radiation and chemotherapy status, IDH1 variant situation, 1p/19q co-deletion status, MGMT promoter methylation rank, TERT expression and TERT mutation position, as well as ATRX mutation status, are just a few of the factors that are taken into consideration.

**Additional file 3: Figure S3.** Stratified survival analysis was performed using Kaplan–Meier survival curves to reveal the clinical and pathological features of the OS classifier based on mtPCDI, including age, gender, tumor grade, chemotherapy and radiotherapy status, Status of the following mutations: IDH1 mutation, MGMT regulator the methylation process 1p/19q co-deletion, TERT expression, TERT change, and ATRX mutation.

**Additional file 4: Figure S4.** The 18 most valuable genes in LGG exhibit genetic, transcriptional, and chemical types. (A) Mutation frequency of 18 most valuable genes in LGG. (B) Variants on chromosomes that are CNV. (C) The gain and loss of CNV between 18 most valuable genes, as well as the frequency of non-CNV. (D) Analysis of differences in the expression of 18 most valuable genes in different mtPCDI groups.

**Additional file 5: Figure S5.** The relationship between genes expression of 18 most valuable and LGG immune subtypes.

**Additional file 6: Figure S6.** The relationship between genes expression of 18 most valuable and LGG molecular subtypes.

**Additional file 7: Figure S7.** Pathway activity between two mtPCDI groups based on ssGSEA algorithm. *p < 0.05; **p < 0.01; ***p < 0.001; ns, no statistical significance.

**Additional file 8: Figure S8.** Chemotherapy regimen predictions. (A) Shown for chemotherapeutic agents with low and high mtPCDI groups using the “oncoPredict”. Similarly, (B) presents for chemotherapeutic agents with low and high mtPCDI groups using the “pRRophetic” program.

**Additional file 9:** The information of 1548 genes related to PCD.

**Additional file 10:** The information of 1136 genes related to mitochondrion.

**Additional file 11:** Intersection genes obtained from Wayne diagram.

**Additional file 12:** Univariate cox regression was used to screen for prognostic related genes.

**Additional file 13:** The performance of 101 predictive models in training and testing cohorts.

**Additional file 14: **Clinical characteristics of high and low mtPCDI risk groups.

**Additional file 15: **Clinical characteristics of C1 and C2 cluster.

**Additional file 16:** Shown for chemotherapeutic agents with low and high mtPCDI groups using the “oncoPredict”.

**Additional file 17:** Shown for chemotherapeutic agents with low and high mtPCDI groups using the “pRRophetic”.

## Data Availability

The original contributions of our study have been reported in the article. Should you have any inquiries regarding our findings, please feel free to direct them to the corresponding author.

## Abbreviations

LGG: Lower-grade glioma
mtPCDI: Mitochondria-associated programmed cell death index
PCD: Programmed cell death
NETs: Neutrophil extracellular trap
TCGA: The Cancer Genome Atlas
CGGA: Chinese Glioma Genome Atlas
GEO: Gene Expression Omnibus
GO: Gene ontology
KEGG: Kyoto Encyclopedia of Genes and Genome
OS: Overall survival
BP: Biological processes
CC: Cellular components
MF: Molecular functions
PAS: Pathway activity score
CSCs: Cancer stem cells
