# Supplementary figures and images for: Integrated machine learning survival framework develops a prognostic model based on inter-crosstalk definition of mitochondrial function and cell death patterns in a large multicenter cohort for lower-grade glioma
Source: J Transl Med. 2023 Sep 2;21:588. doi: 10.1186/s12967-023-04468-x (PMC10474752; doi:10.1186/s12967-023-04468-x)

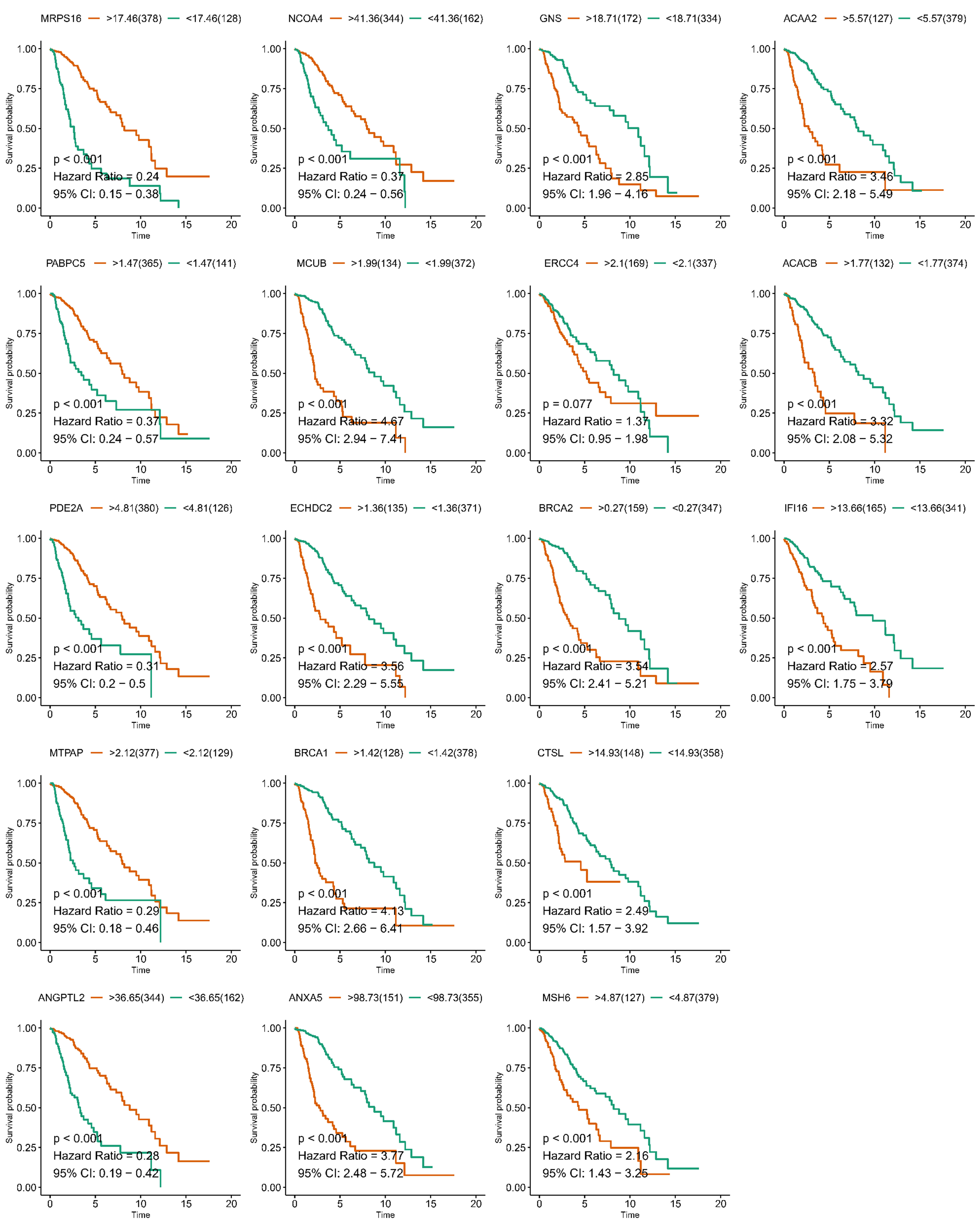

Supplement: Supplementary file 1 — Additional file 1: Figure S1. Kaplan–Meier mortality line was calculated of OS between patients with high or low expression of 18 most valuable. [file 12967_2023_4468_MOESM1_ESM.tif]

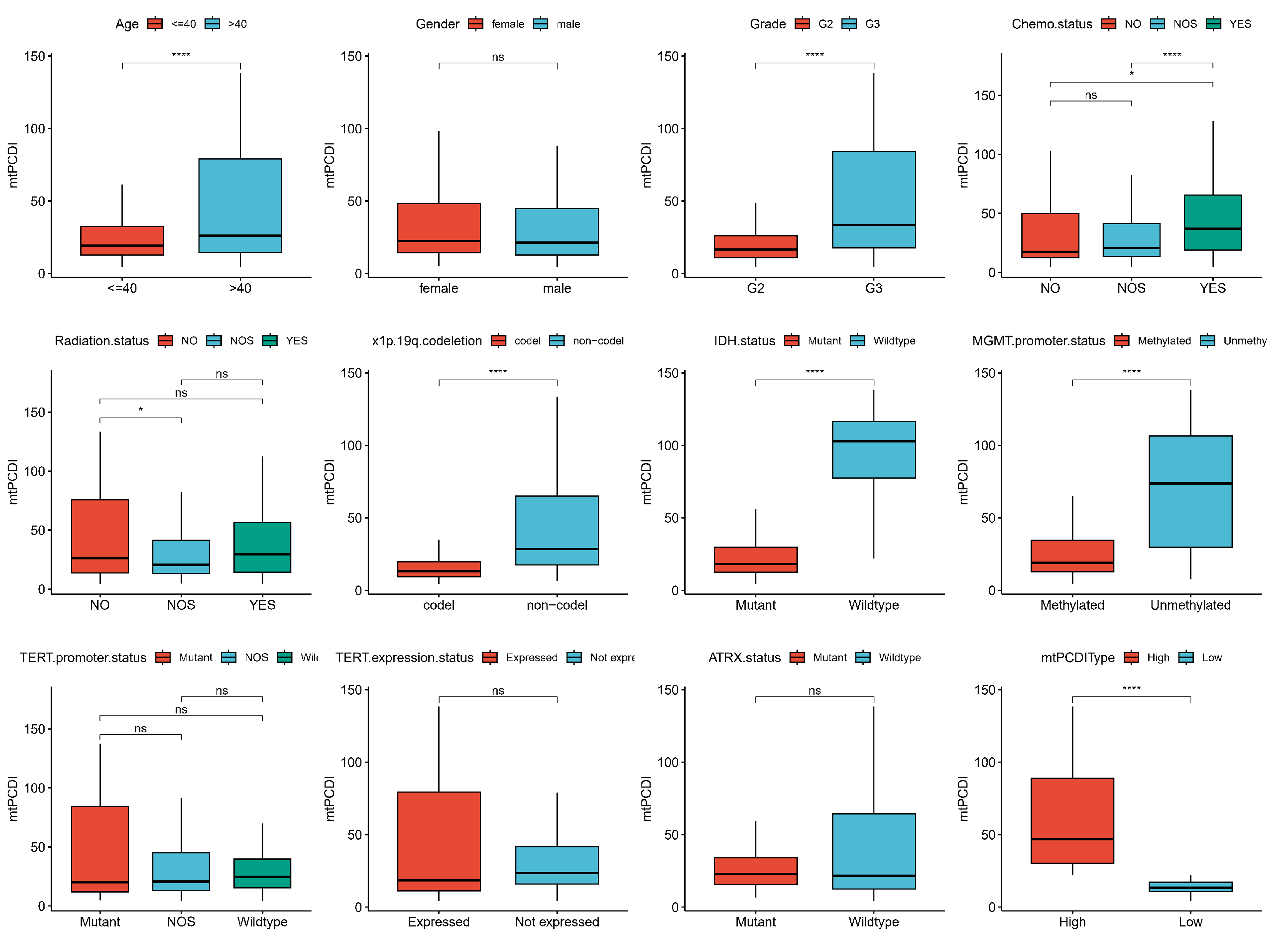

Supplement: Supplementary file 2 — Additional file 2: Figure S2. Differential analysis of mtPCDI correlation between clinical and pathological variables among different subgroups. Age, gender, tumor levels, radiation and chemotherapy status, IDH1 variant situation, 1p/19q co-deletion status, MGMT promoter methylation rank, TERT expression and TERT mutation position, as well as ATRX mutation status, are just a few of the factors that are taken into consideration. [file 12967_2023_4468_MOESM2_ESM.tif]

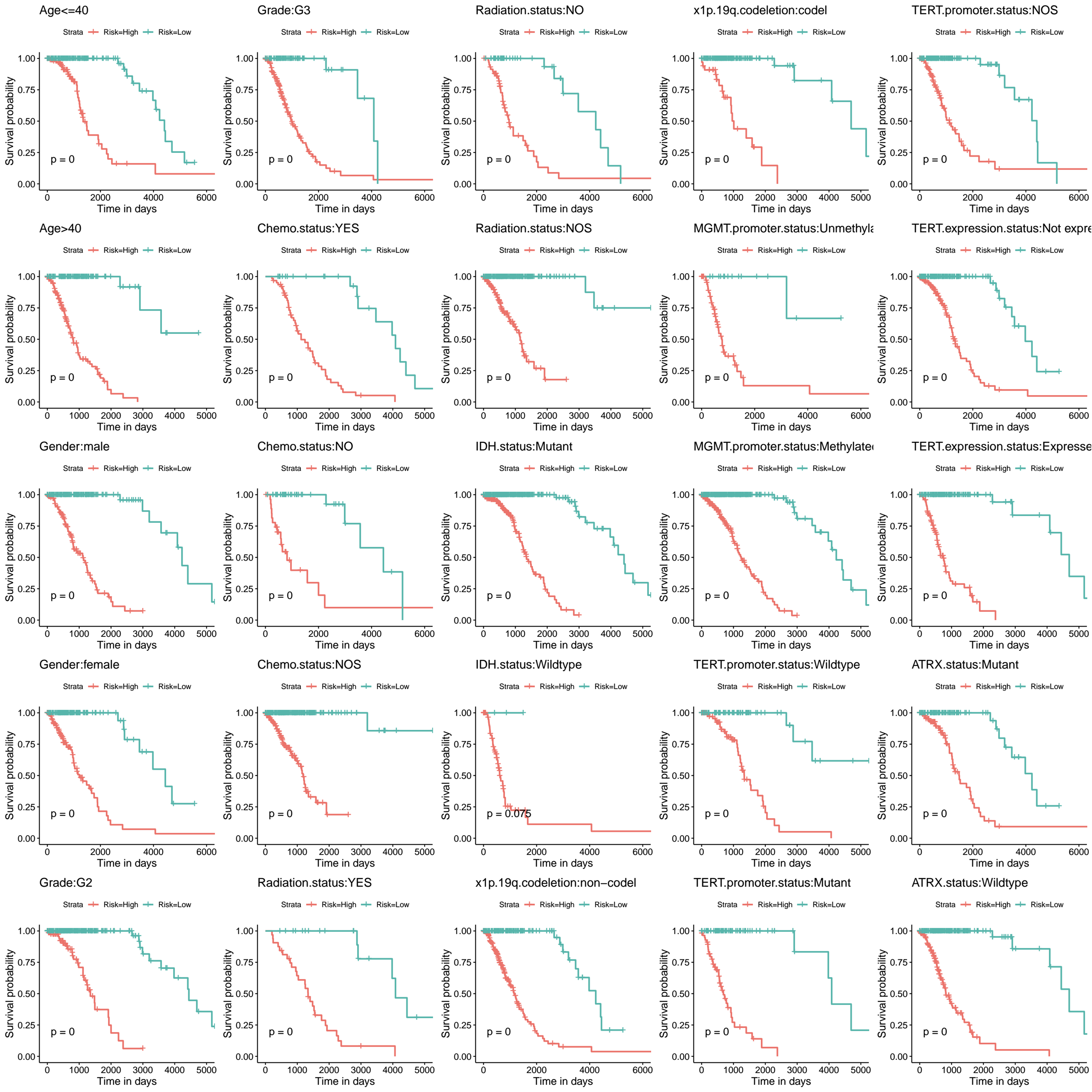

Supplement: Supplementary file 3 — Additional file 3: Figure S3. Stratified survival analysis was performed using Kaplan–Meier survival curves to reveal the clinical and pathological features of the OS classifier based on mtPCDI, including age, gender, tumor grade, chemotherapy and radiotherapy status, Status of the following mutations: IDH1 mutation, MGMT regulator the methylation process 1p/19q co-deletion, TERT expression, TERT change, and ATRX mutation. [file 12967_2023_4468_MOESM3_ESM.tif]

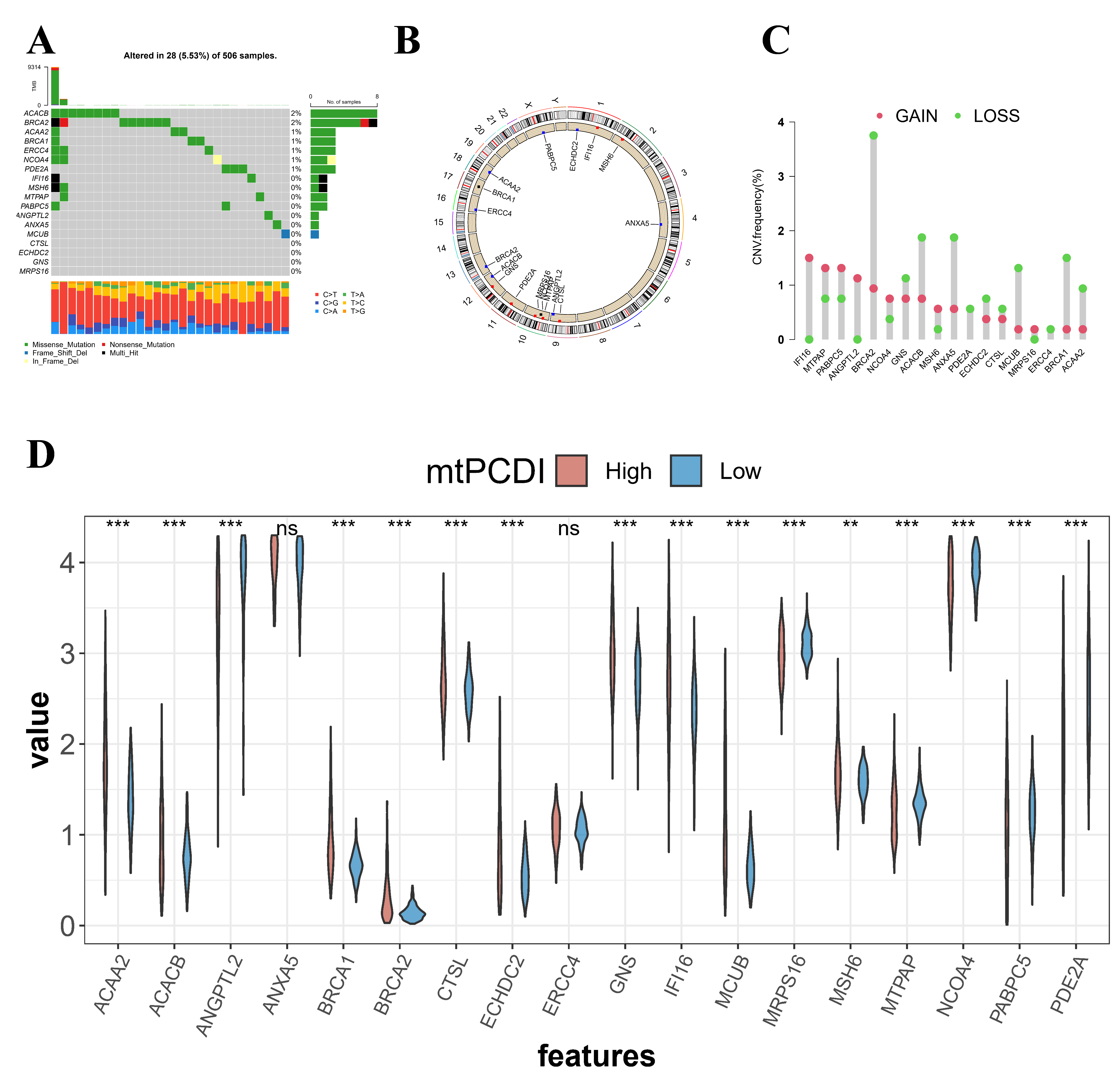

Supplement: Supplementary file 4 — Additional file 4: Figure S4. The 18 most valuable genes in LGG exhibit genetic, transcriptional, and chemical types. (A) Mutation frequency of 18 most valuable genes in LGG. (B) Variants on chromosomes that are CNV. (C) The gain and loss of CNV between 18 most valuable genes, as well as the frequency of non-CNV. (D) Analysis of differences in the expression of 18 most valuable genes in different mtPCDI groups. [file 12967_2023_4468_MOESM4_ESM.tif]

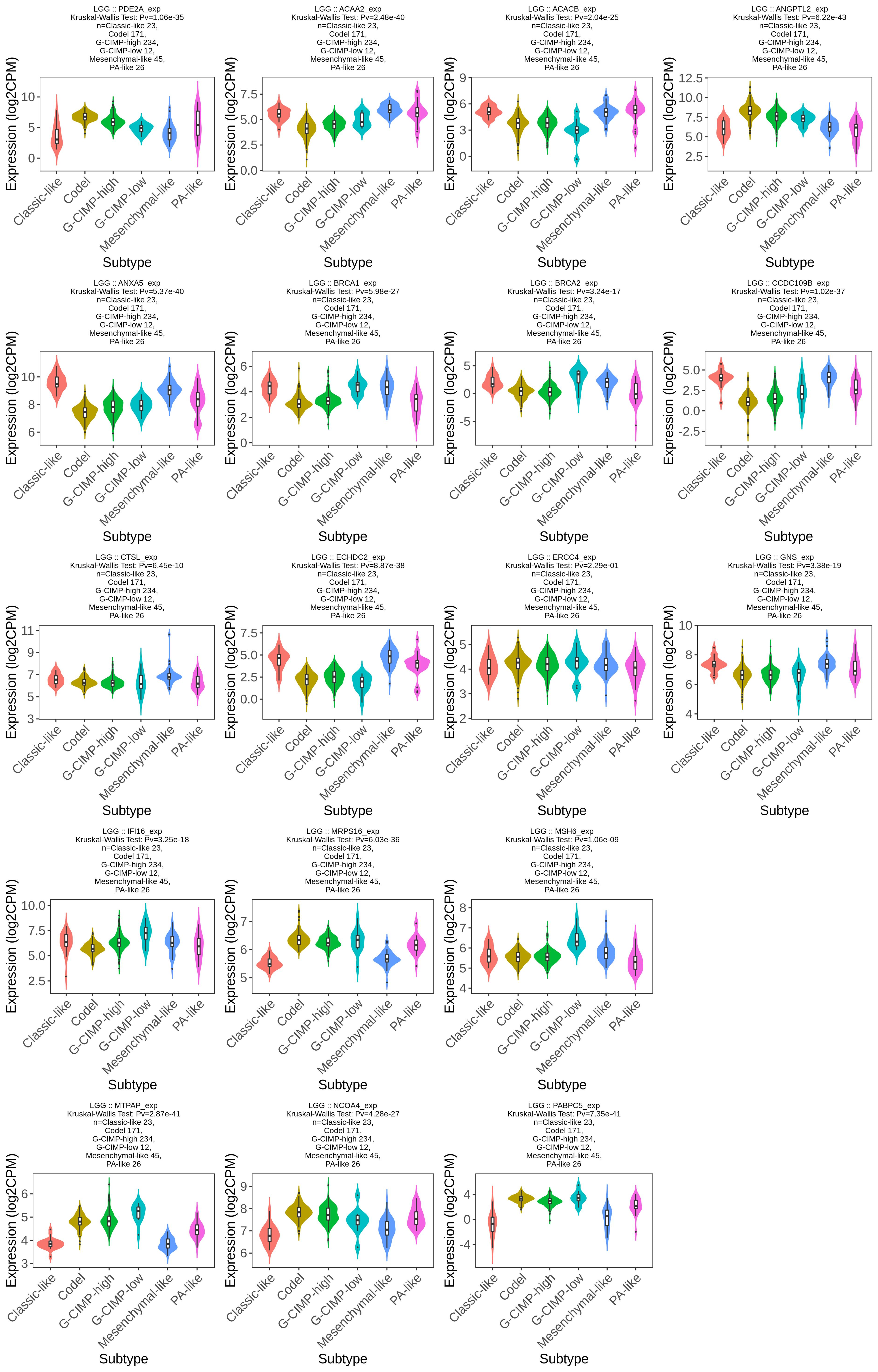

Supplement: Supplementary file 5 — Additional file 5: Figure S5. The relationship between genes expression of 18 most valuable and LGG immune subtypes. [file 12967_2023_4468_MOESM5_ESM.tif]

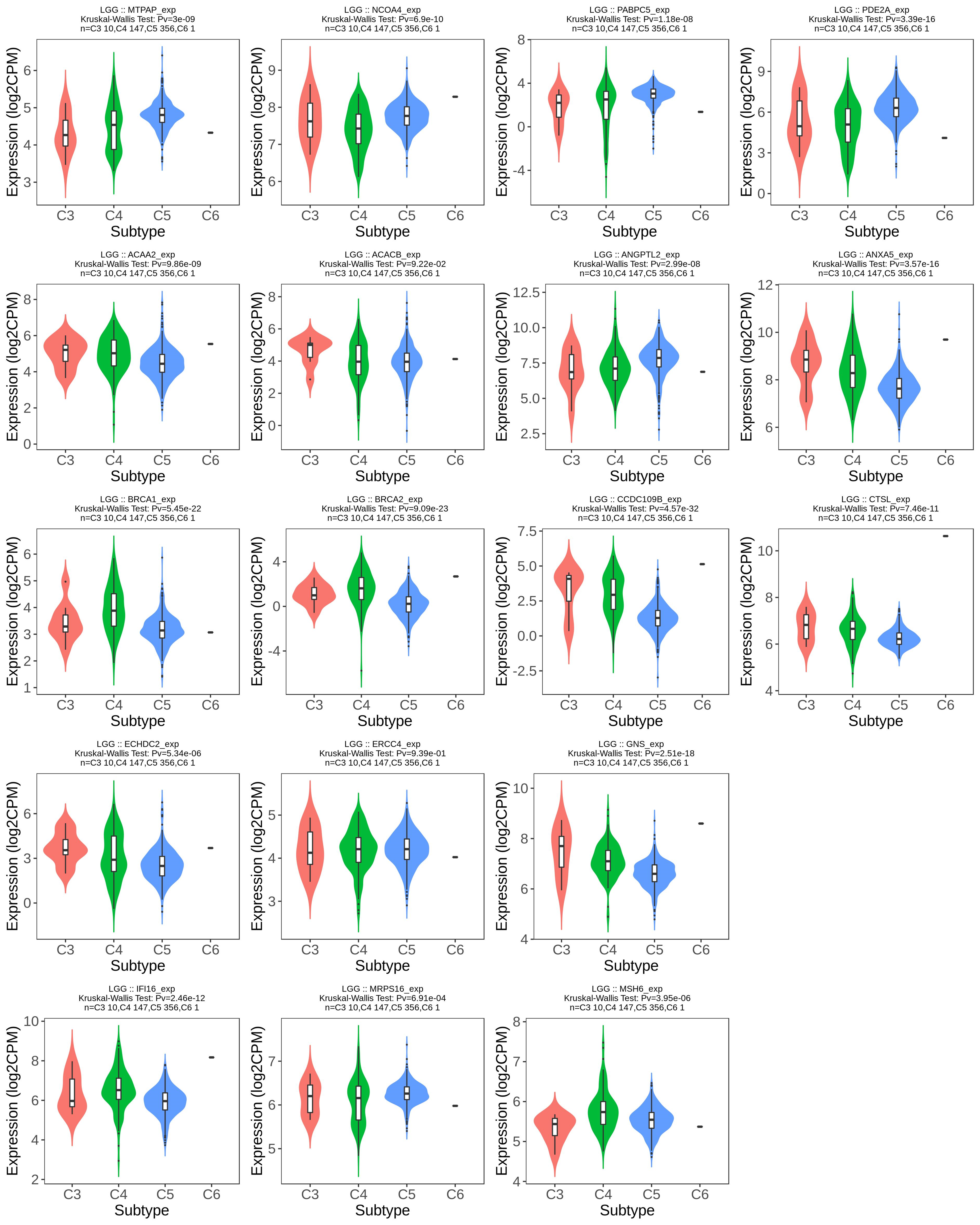

Supplement: Supplementary file 6 — Additional file 6: Figure S6. The relationship between genes expression of 18 most valuable and LGG molecular subtypes. [file 12967_2023_4468_MOESM6_ESM.tif]
